# Supplementary material for: Membrane curvature initiates Cdc42-FBP17-N-WASP clustering and actin nucleation
Source: EMBO J. 2026 Jan 3;45(3):953–86. doi: 10.1038/s44318-025-00677-w (PMC12864879; doi:10.1038/s44318-025-00677-w)
Supplement: Supplementary file 1 — Table EV1 [file 44318_2025_677_MOESM1_ESM.docx]

**Table EV1. Table of simulation parameters used in MEDYAN to simulate dendritic actin network. The mesoscopic rates used are shown within square brackets.**

| **Parameter** | **Value** | **Comment/Reference** |
| --- | --- | --- |
| **Geometric parameters** |  |  |
| Compartment size $(L_{comp)}$ | 500 nm | (Popov *et al.*, 2016) |
| Number of compartments in each dimension $(N_{x}\times N_{y}\times N_{z})$ | $16\times16\times1$ | - |
| Length of filament segment cylinder $L_{cyl}$ | 27 nm (10 subunits) | - |
| Binding sites per cylinder $N_{b,site}$ | 1 | - |
| **Diffusion rates** |  |  |
| G-actin | $D_{actin}=20 \mu m^{2}/s$  $k_{diff,actin}= 80 /s$ | (Popov *et al.*, 2016) |
| N-WASP | $D_{NWASP}=0 \mu m^{2}/s$ | a |
| Arp2/3 | $kdiff,Arp2/3$  $=k_{diff,actin}/10$ | (Chandrasekaran *et al*, 2022a; Chandrasekaran *et al*, 2022b) |
| **Kinetic rate constants** |  |  |
| Actin polymerization at plus ends ($k_{poly, +}$) | $11.6 (\mu M,s)^{-1} [0.154 s^{-1}]$ | (Fujiwara *et al*, 2007) |
| Actin depolymerization at plus ends ($k_{depoly, +}$) | $1.4 s^{-1}$ | (Fujiwara *et al.*, 2007) |
| Actin polymerization at minus ends ($k_{poly, -}$) | $1.3 (\mu M,s)^{-1} [0.017 s^{-1}]$ | (Fujiwara *et al.*, 2007) |
| Actin polymerization at minus ends ($k_{depoly, -}$) | $0.8 s^{-1}$ | (Fujiwara *et al.*, 2007) |
| Arp2/3 activation rate ($k_{Arp2/3,activate}$) | $0.075, 0.0075, 0.00075$  $(\mu M.s)^{-1}$ [$10^{-3}, 10^{-4}, 10^{-5}] s^{-1}$ | In this study |
| Arp2/3 deactivation rate ($k_{Arp2/3,deactivate}$) | $0.01s^{-1}$ | In this study |
| Arp2/3 binding rate ($k_{Arp2/3,bind}$) | $10.0 (\mu M^{2}.s)^{-1}[0.0017 {/s}^{-1}]$ | ODE model in (Chandrasekaran *et al.*, 2022a) |
| Arp2/3 unbinding rate (  $k_{Arp2/3,unbind}$) | $0.02 s^{-1}$ | (Mahaffy & Pollard, 2006) |
| **Concentrations** |  |  |
| Total Actin | $1 \mu M$ (19273 molecules) | ^-^ |
| Total N-WASP | $1 \mu M$ (19273 molecules) | ^-^ |
| Total Arp2/3 | 10 nM (198 molecules) |  |
| **Mechanochemical constants** |  |  |
| Arp2/3 unbinding force | $F_{branch,unbind}=6pN$ | (Fujiwara *et al*, 2002) |
| Characteristic force of Brownian ratchet | $F_{0, ratchet}=$ 1.5 pN | (Footer *et al*, 2007) |
| **Mechanical constants** |  |  |
| Actin filament stretching constant $(k_{str}),$ and rest length $l_{0}$ | $100pN/nm$  $l_{0}=L_{cyl}$ | (Popov *et al.*, 2016) |
| Actin filament bending energy ($k_{bend}$) | $2690pN.nm$ | (Gittes *et al*, 1993) |
| Boundary repulsion energy $(\epsilon_{boundary})$ | $10k_{B}T$ | - |
| Boundary repulsion screening length ($\lambda$) | 2.7 nm | - |
| Arp2/3 stretching constant $(k_{branch,.stretch})$, and rest length $L_{0}$ | $k_{branch, stretch}=$100 pN/nm,  $L_{0}=6 nm$ | (Chandrasekaran *et al.*, 2022b) |
| Arp2/3 bending constant, I ($k_{branch,.bend,I}$), equilibrium angle | $10 pN.nm, \pi/2$ | (Chandrasekaran *et al.*, 2022b) |
| Arp2/3 bending constant, II (  $k_{branch,.bendi,I}$), equilibrium angle | $20 pN.nm, 70^{o}$ | (Chandrasekaran *et al.*, 2022b) |
| Arp2/3 dihedral constant ($k_{branch, dihedral}$) | $10 pN.nm$ | (Chandrasekaran *et al.*, 2022b) |
| **Minimization parameters** |  |  |
| Time scale of chemical steps | 5 ms |  |
| Force tolerance for mechanical minimization | 10 pN |  |
| Seed filaments |  |  |
| Number | 10 |  |
| Length | 27 nm |  |
| **Simulation parameters** |  |  |
| Total time | 400 s |  |
| Replicates | 3 |  |

^a^We assume N-WASP to be membrane-bound and assume that the N-WASP does not diffuse during the timescale of the simulation
