# Supplementary figures and images for: Membrane curvature initiates Cdc42-FBP17-N-WASP clustering and actin nucleation (part 1 of 2)
Source: EMBO J. 2026 Jan 3;45(3):953–86. doi: 10.1038/s44318-025-00677-w (PMC12864879; doi:10.1038/s44318-025-00677-w)

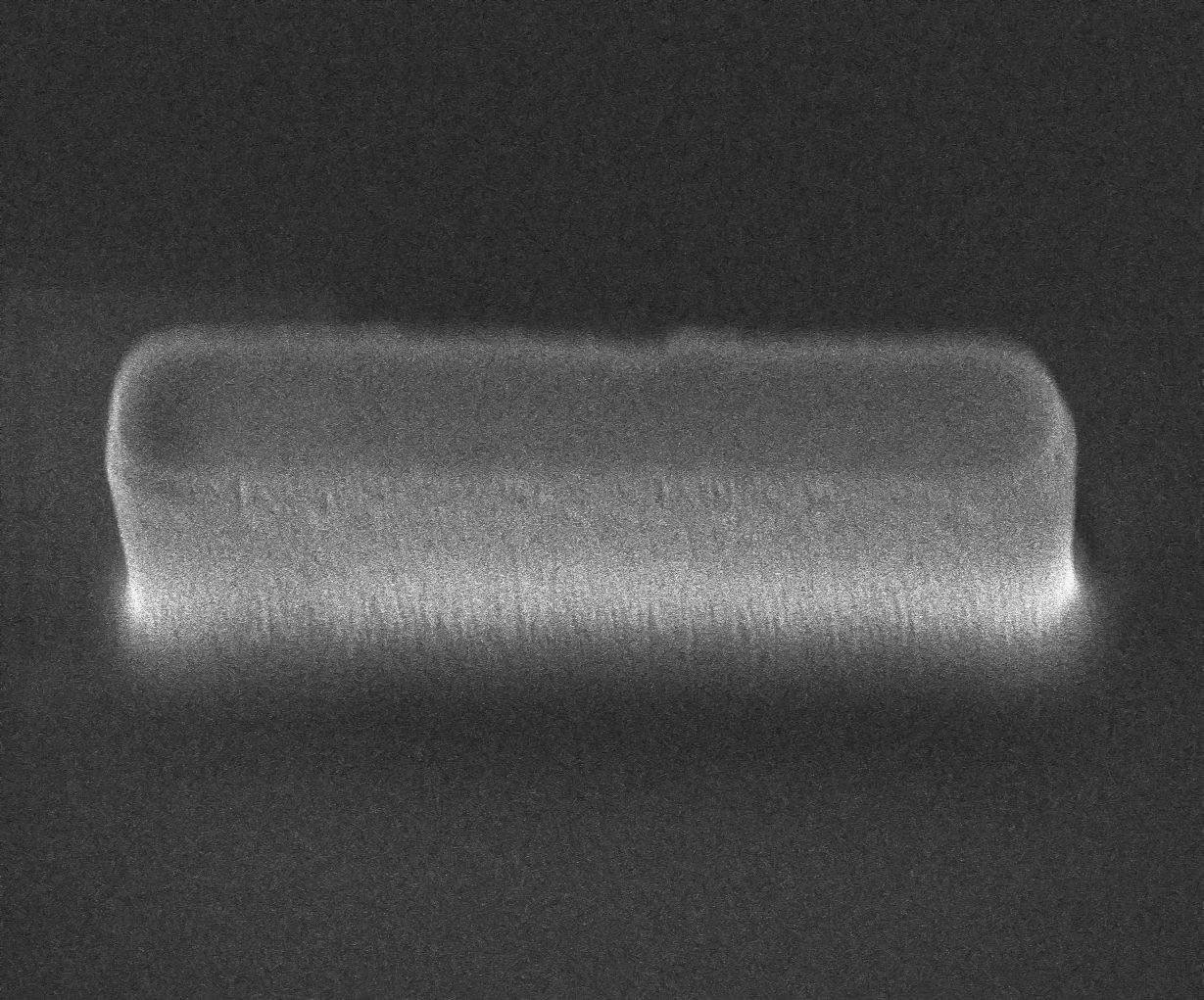

Supplement: Supplementary file 9 — Source data Fig. 1 [file 44318_2025_677_MOESM9_ESM.zip › Figure 1/1B/Figure 1B_SEM image for nanobar.tif]

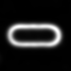

Supplement: Supplementary file 9 — Source data Fig. 1 [file 44318_2025_677_MOESM9_ESM.zip › Figure 1/1D/1D_FBP17_16 bit_stack.tif]

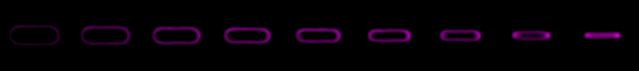

Supplement: Supplementary file 9 — Source data Fig. 1 [file 44318_2025_677_MOESM9_ESM.zip › Figure 1/1D/1D_FBP17_Montage.tif]

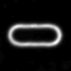

Supplement: Supplementary file 9 — Source data Fig. 1 [file 44318_2025_677_MOESM9_ESM.zip › Figure 1/1D/1D_N-WASP_16 bit_stack.tif]

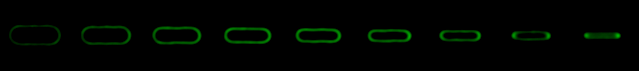

Supplement: Supplementary file 9 — Source data Fig. 1 [file 44318_2025_677_MOESM9_ESM.zip › Figure 1/1D/1D_N-WASP_Montage.tif]

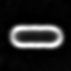

Supplement: Supplementary file 9 — Source data Fig. 1 [file 44318_2025_677_MOESM9_ESM.zip › Figure 1/1D/1D_SLB_16 bit_stack.tif]

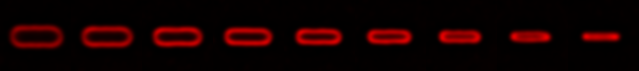

Supplement: Supplementary file 9 — Source data Fig. 1 [file 44318_2025_677_MOESM9_ESM.zip › Figure 1/1D/1D_SLB_Montage.tif]

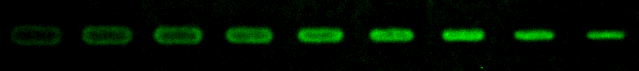

Supplement: Supplementary file 9 — Source data Fig. 1 [file 44318_2025_677_MOESM9_ESM.zip › Figure 1/1G/1G_N-WASP control Montage.tif]

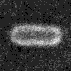

Supplement: Supplementary file 9 — Source data Fig. 1 [file 44318_2025_677_MOESM9_ESM.zip › Figure 1/1G/1G_N-WASP control_16bit stack.tif]

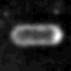

Supplement: Supplementary file 10 — Source data Fig. 2 [file 44318_2025_677_MOESM10_ESM.zip › Figure 2/2A/2A_FBP17 stack/1000.tif]

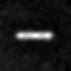

Supplement: Supplementary file 10 — Source data Fig. 2 [file 44318_2025_677_MOESM10_ESM.zip › Figure 2/2A/2A_FBP17 stack/200.tif]

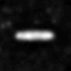

Supplement: Supplementary file 10 — Source data Fig. 2 [file 44318_2025_677_MOESM10_ESM.zip › Figure 2/2A/2A_FBP17 stack/300.tif]

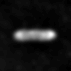

Supplement: Supplementary file 10 — Source data Fig. 2 [file 44318_2025_677_MOESM10_ESM.zip › Figure 2/2A/2A_FBP17 stack/400.tif]

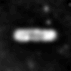

Supplement: Supplementary file 10 — Source data Fig. 2 [file 44318_2025_677_MOESM10_ESM.zip › Figure 2/2A/2A_FBP17 stack/500.tif]

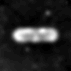

Supplement: Supplementary file 10 — Source data Fig. 2 [file 44318_2025_677_MOESM10_ESM.zip › Figure 2/2A/2A_FBP17 stack/600.tif]

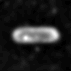

Supplement: Supplementary file 10 — Source data Fig. 2 [file 44318_2025_677_MOESM10_ESM.zip › Figure 2/2A/2A_FBP17 stack/700.tif]

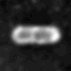

Supplement: Supplementary file 10 — Source data Fig. 2 [file 44318_2025_677_MOESM10_ESM.zip › Figure 2/2A/2A_FBP17 stack/800.tif]

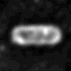

Supplement: Supplementary file 10 — Source data Fig. 2 [file 44318_2025_677_MOESM10_ESM.zip › Figure 2/2A/2A_FBP17 stack/900.tif]

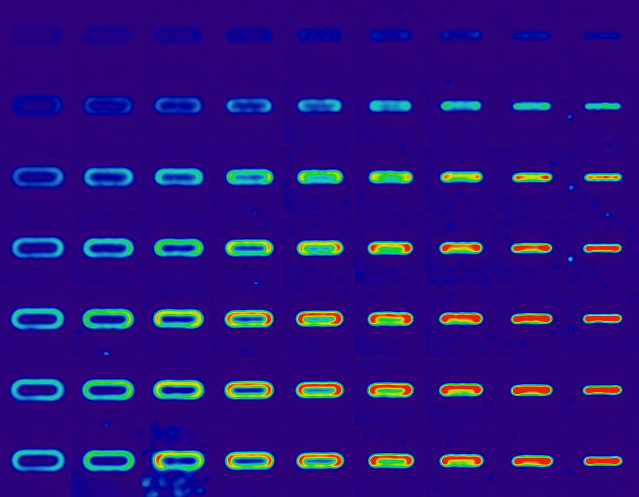

Supplement: Supplementary file 10 — Source data Fig. 2 [file 44318_2025_677_MOESM10_ESM.zip › Figure 2/2A/2A_FBP17 titration.tif]

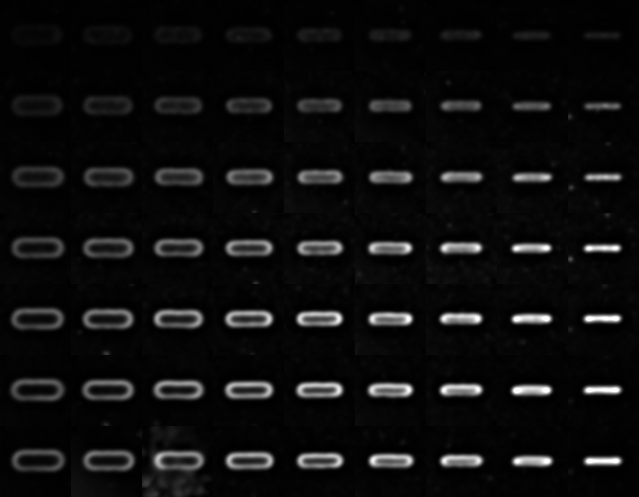

Supplement: Supplementary file 10 — Source data Fig. 2 [file 44318_2025_677_MOESM10_ESM.zip › Figure 2/2A/2A_FBP17 titration_16bit.tif]

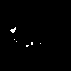

Supplement: Supplementary file 10 — Source data Fig. 2 [file 44318_2025_677_MOESM10_ESM.zip › Figure 2/2C/2C_N-WASP stack/1000.tif]

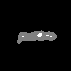

Supplement: Supplementary file 10 — Source data Fig. 2 [file 44318_2025_677_MOESM10_ESM.zip › Figure 2/2C/2C_N-WASP stack/200.tif]

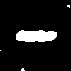

Supplement: Supplementary file 10 — Source data Fig. 2 [file 44318_2025_677_MOESM10_ESM.zip › Figure 2/2C/2C_N-WASP stack/300.tif]

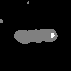

Supplement: Supplementary file 10 — Source data Fig. 2 [file 44318_2025_677_MOESM10_ESM.zip › Figure 2/2C/2C_N-WASP stack/400.tif]

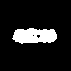

Supplement: Supplementary file 10 — Source data Fig. 2 [file 44318_2025_677_MOESM10_ESM.zip › Figure 2/2C/2C_N-WASP stack/500.tif]

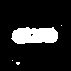

Supplement: Supplementary file 10 — Source data Fig. 2 [file 44318_2025_677_MOESM10_ESM.zip › Figure 2/2C/2C_N-WASP stack/600.tif]

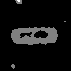

Supplement: Supplementary file 10 — Source data Fig. 2 [file 44318_2025_677_MOESM10_ESM.zip › Figure 2/2C/2C_N-WASP stack/700.tif]

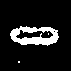

Supplement: Supplementary file 10 — Source data Fig. 2 [file 44318_2025_677_MOESM10_ESM.zip › Figure 2/2C/2C_N-WASP stack/800.tif]

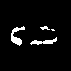

Supplement: Supplementary file 10 — Source data Fig. 2 [file 44318_2025_677_MOESM10_ESM.zip › Figure 2/2C/2C_N-WASP stack/900.tif]

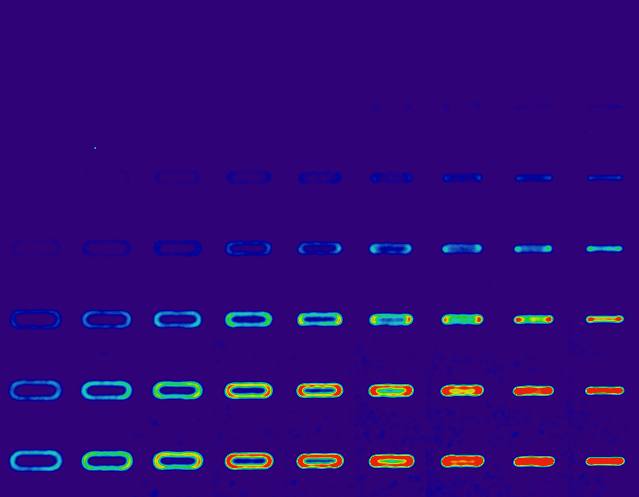

Supplement: Supplementary file 10 — Source data Fig. 2 [file 44318_2025_677_MOESM10_ESM.zip › Figure 2/2C/2C_N-WASP_Montage.tif]

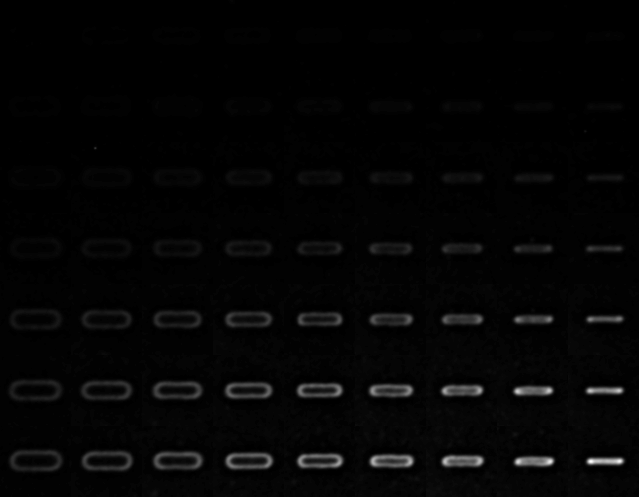

Supplement: Supplementary file 10 — Source data Fig. 2 [file 44318_2025_677_MOESM10_ESM.zip › Figure 2/2C/2C_N-WASP_Montage_16bit.tif]

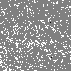

Supplement: Supplementary file 10 — Source data Fig. 2 [file 44318_2025_677_MOESM10_ESM.zip › Figure 2/2E/2E_N-WASP control_16bit_stack/1000.tif]

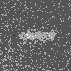

Supplement: Supplementary file 10 — Source data Fig. 2 [file 44318_2025_677_MOESM10_ESM.zip › Figure 2/2E/2E_N-WASP control_16bit_stack/200.tif]

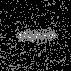

Supplement: Supplementary file 10 — Source data Fig. 2 [file 44318_2025_677_MOESM10_ESM.zip › Figure 2/2E/2E_N-WASP control_16bit_stack/300.tif]

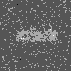

Supplement: Supplementary file 10 — Source data Fig. 2 [file 44318_2025_677_MOESM10_ESM.zip › Figure 2/2E/2E_N-WASP control_16bit_stack/400.tif]

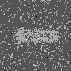

Supplement: Supplementary file 10 — Source data Fig. 2 [file 44318_2025_677_MOESM10_ESM.zip › Figure 2/2E/2E_N-WASP control_16bit_stack/500.tif]

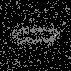

Supplement: Supplementary file 10 — Source data Fig. 2 [file 44318_2025_677_MOESM10_ESM.zip › Figure 2/2E/2E_N-WASP control_16bit_stack/600.tif]

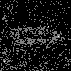

Supplement: Supplementary file 10 — Source data Fig. 2 [file 44318_2025_677_MOESM10_ESM.zip › Figure 2/2E/2E_N-WASP control_16bit_stack/700.tif]

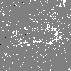

Supplement: Supplementary file 10 — Source data Fig. 2 [file 44318_2025_677_MOESM10_ESM.zip › Figure 2/2E/2E_N-WASP control_16bit_stack/800.tif]

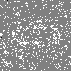

Supplement: Supplementary file 10 — Source data Fig. 2 [file 44318_2025_677_MOESM10_ESM.zip › Figure 2/2E/2E_N-WASP control_16bit_stack/900.tif]

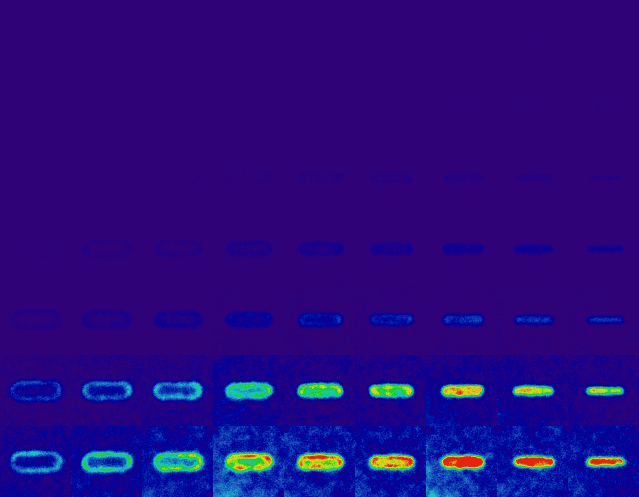

Supplement: Supplementary file 10 — Source data Fig. 2 [file 44318_2025_677_MOESM10_ESM.zip › Figure 2/2E/2E_N-WASP_control_Montage.tif]

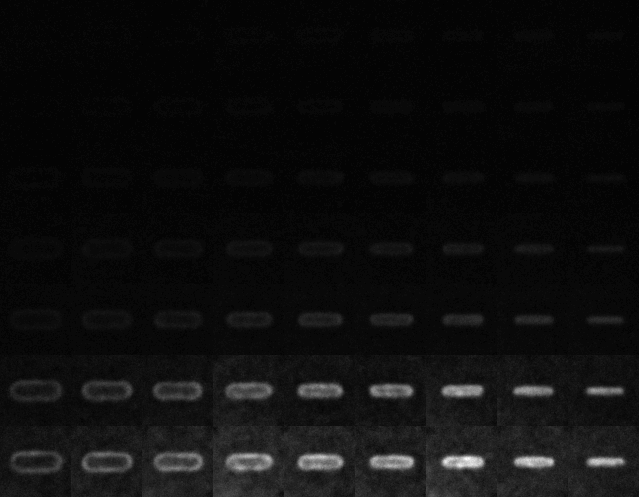

Supplement: Supplementary file 10 — Source data Fig. 2 [file 44318_2025_677_MOESM10_ESM.zip › Figure 2/2E/2E_N-WASP_control_Montage_16bit.tif]

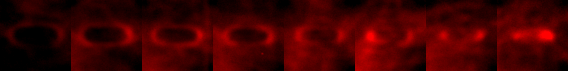

Supplement: Supplementary file 11 — Source data Fig. 3 [file 44318_2025_677_MOESM11_ESM.zip › Figure 3/3B/3B_DMSO/3B_Lifeact-mApple_DMSO.tif]

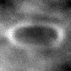

Supplement: Supplementary file 11 — Source data Fig. 3 [file 44318_2025_677_MOESM11_ESM.zip › Figure 3/3B/3B_DMSO/3B_Lifeact-mApple_DMSO_16bit_Stack.tif]

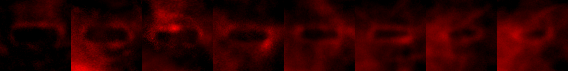

Supplement: Supplementary file 11 — Source data Fig. 3 [file 44318_2025_677_MOESM11_ESM.zip › Figure 3/3B/3B_LatA/3B_Lifeact-mApple_LatA.tif]

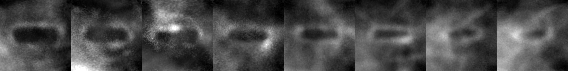

Supplement: Supplementary file 11 — Source data Fig. 3 [file 44318_2025_677_MOESM11_ESM.zip › Figure 3/3B/3B_LatA/3B_Lifeact-mApple_LatA_16bit.tif]

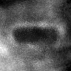

Supplement: Supplementary file 11 — Source data Fig. 3 [file 44318_2025_677_MOESM11_ESM.zip › Figure 3/3B/3B_LatA/3B_Lifeact-mApple_LatA_16bit_Stack.tif]

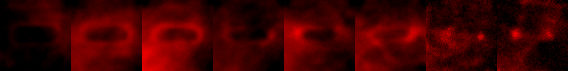

Supplement: Supplementary file 11 — Source data Fig. 3 [file 44318_2025_677_MOESM11_ESM.zip › Figure 3/3B/3B_Washout/3B_Lifeact-mApple_Washout.tif]

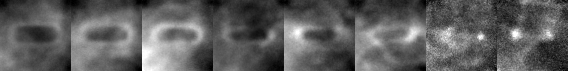

Supplement: Supplementary file 11 — Source data Fig. 3 [file 44318_2025_677_MOESM11_ESM.zip › Figure 3/3B/3B_Washout/3B_Lifeact-mApple_Washout_16bit.tif]

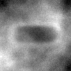

Supplement: Supplementary file 11 — Source data Fig. 3 [file 44318_2025_677_MOESM11_ESM.zip › Figure 3/3B/3B_Washout/3B_Lifeact-mApple_Washout_16bit_Stack.tif]

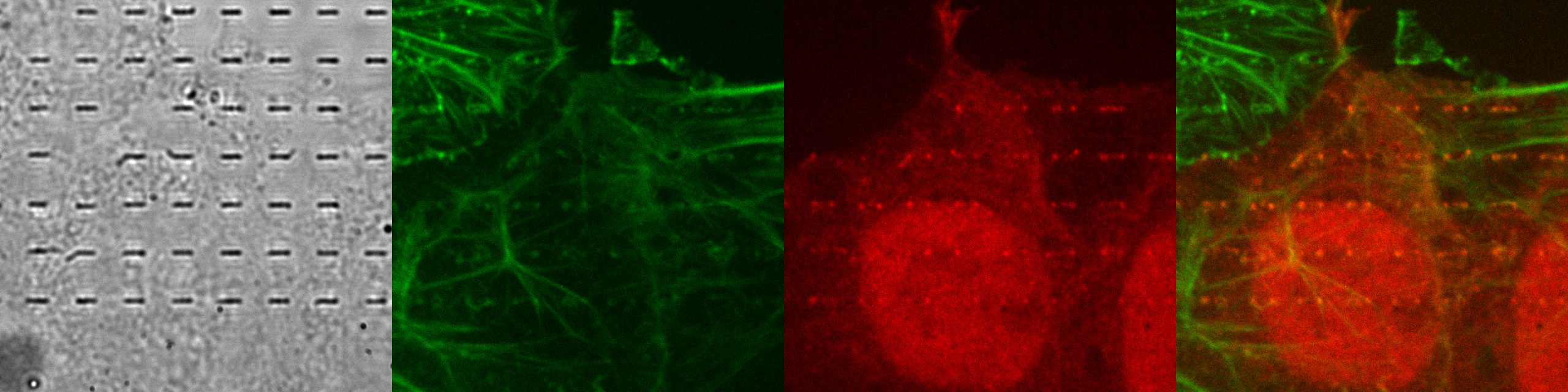

Supplement: Supplementary file 11 — Source data Fig. 3 [file 44318_2025_677_MOESM11_ESM.zip › Figure 3/3E/3E_Montage_20uM ML141.tif]

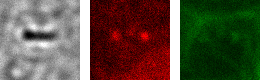

Supplement: Supplementary file 11 — Source data Fig. 3 [file 44318_2025_677_MOESM11_ESM.zip › Figure 3/3E/3E_Montage_20uM ML141_80x80.tif]

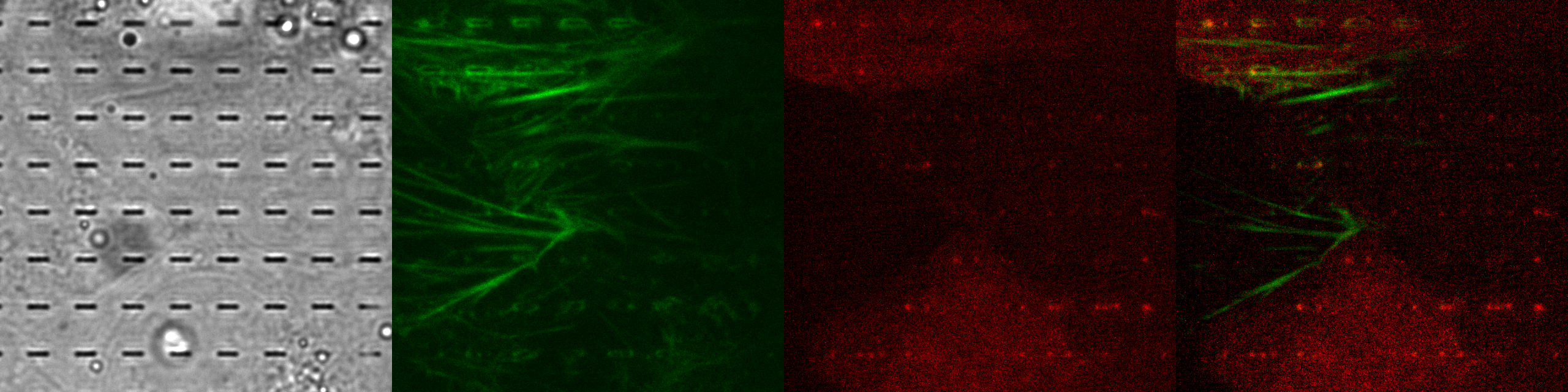

Supplement: Supplementary file 11 — Source data Fig. 3 [file 44318_2025_677_MOESM11_ESM.zip › Figure 3/3E/3E_Montage_50uM ML141.tif]

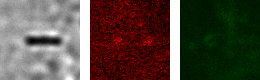

Supplement: Supplementary file 11 — Source data Fig. 3 [file 44318_2025_677_MOESM11_ESM.zip › Figure 3/3E/3E_Montage_50uM ML141_80x80.tif]

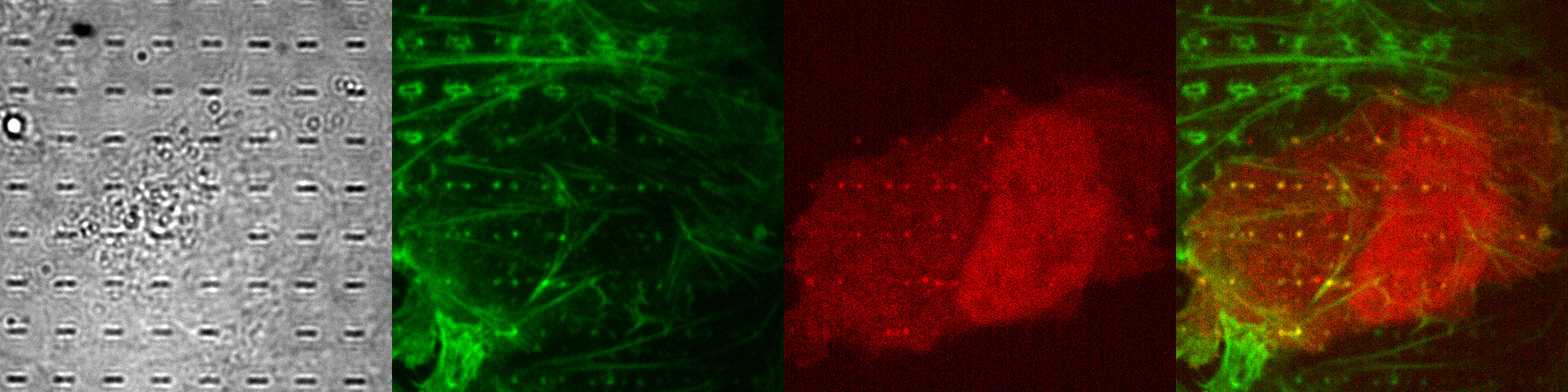

Supplement: Supplementary file 11 — Source data Fig. 3 [file 44318_2025_677_MOESM11_ESM.zip › Figure 3/3E/3E_Montage_DMSO.tif]

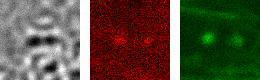

Supplement: Supplementary file 11 — Source data Fig. 3 [file 44318_2025_677_MOESM11_ESM.zip › Figure 3/3E/3E_Montage_DMSO_80x80.tif]

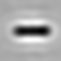

Supplement: Supplementary file 11 — Source data Fig. 3 [file 44318_2025_677_MOESM11_ESM.zip › Figure 3/3E/Averaged nanobar images/20uM ML141_All_MaskAvg-267-w1BF-1.tif]

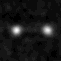

Supplement: Supplementary file 11 — Source data Fig. 3 [file 44318_2025_677_MOESM11_ESM.zip › Figure 3/3E/Averaged nanobar images/20uM ML141_All_MaskAvg-267-w2TXR-1.tif]

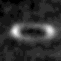

Supplement: Supplementary file 11 — Source data Fig. 3 [file 44318_2025_677_MOESM11_ESM.zip › Figure 3/3E/Averaged nanobar images/20uM ML141_All_MaskAvg-267-w3GFP-1.tif]

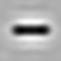

Supplement: Supplementary file 11 — Source data Fig. 3 [file 44318_2025_677_MOESM11_ESM.zip › Figure 3/3E/Averaged nanobar images/50uM ML141_All_MaskAvg-347-w1BF-1.tif]

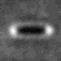

Supplement: Supplementary file 11 — Source data Fig. 3 [file 44318_2025_677_MOESM11_ESM.zip › Figure 3/3E/Averaged nanobar images/50uM ML141_All_MaskAvg-347-w2TXR-1.tif]

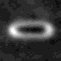

Supplement: Supplementary file 11 — Source data Fig. 3 [file 44318_2025_677_MOESM11_ESM.zip › Figure 3/3E/Averaged nanobar images/50uM ML141_All_MaskAvg-347-w3GFP-1.tif]

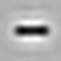

Supplement: Supplementary file 11 — Source data Fig. 3 [file 44318_2025_677_MOESM11_ESM.zip › Figure 3/3E/Averaged nanobar images/DMSO_All_MaskAvg-317-w1BF-1.tif]

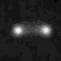

Supplement: Supplementary file 11 — Source data Fig. 3 [file 44318_2025_677_MOESM11_ESM.zip › Figure 3/3E/Averaged nanobar images/DMSO_All_MaskAvg-317-w2TXR-1.tif]

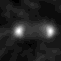

Supplement: Supplementary file 11 — Source data Fig. 3 [file 44318_2025_677_MOESM11_ESM.zip › Figure 3/3E/Averaged nanobar images/DMSO_All_MaskAvg-317-w3GFP-1.tif]

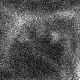

Supplement: Supplementary file 11 — Source data Fig. 3 [file 44318_2025_677_MOESM11_ESM.zip › Figure 3/3E/Original nanobar images/20uM ML141_Actin.tif]

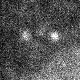

Supplement: Supplementary file 11 — Source data Fig. 3 [file 44318_2025_677_MOESM11_ESM.zip › Figure 3/3E/Original nanobar images/20uM ML141_N-WASP.tif]

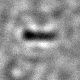

Supplement: Supplementary file 11 — Source data Fig. 3 [file 44318_2025_677_MOESM11_ESM.zip › Figure 3/3E/Original nanobar images/20uM ML141_NANOBAR.tif]

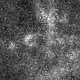

Supplement: Supplementary file 11 — Source data Fig. 3 [file 44318_2025_677_MOESM11_ESM.zip › Figure 3/3E/Original nanobar images/50uM ML141_actin.tif]

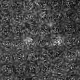

Supplement: Supplementary file 11 — Source data Fig. 3 [file 44318_2025_677_MOESM11_ESM.zip › Figure 3/3E/Original nanobar images/50uM ML141_N-WASP.tif]

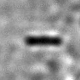

Supplement: Supplementary file 11 — Source data Fig. 3 [file 44318_2025_677_MOESM11_ESM.zip › Figure 3/3E/Original nanobar images/50uM ML141_NANOBAR.tif]

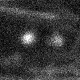

Supplement: Supplementary file 11 — Source data Fig. 3 [file 44318_2025_677_MOESM11_ESM.zip › Figure 3/3E/Original nanobar images/DMSO_Actin.tif]

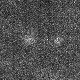

Supplement: Supplementary file 11 — Source data Fig. 3 [file 44318_2025_677_MOESM11_ESM.zip › Figure 3/3E/Original nanobar images/DMSO_N-WASP.tif]

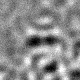

Supplement: Supplementary file 11 — Source data Fig. 3 [file 44318_2025_677_MOESM11_ESM.zip › Figure 3/3E/Original nanobar images/DMSO_NANOBAR.tif]

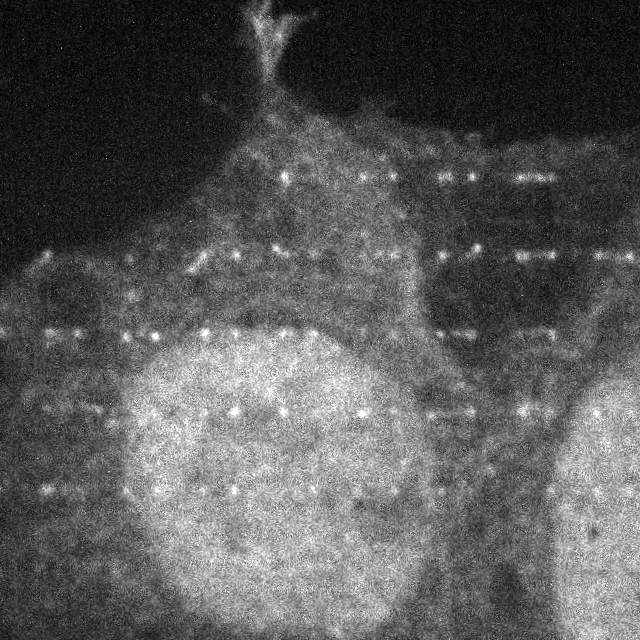

Supplement: Supplementary file 11 — Source data Fig. 3 [file 44318_2025_677_MOESM11_ESM.zip › Figure 3/3E/U2OS cell Images/20uM ML141_74_w1CSU mCherry_640x640.tif]

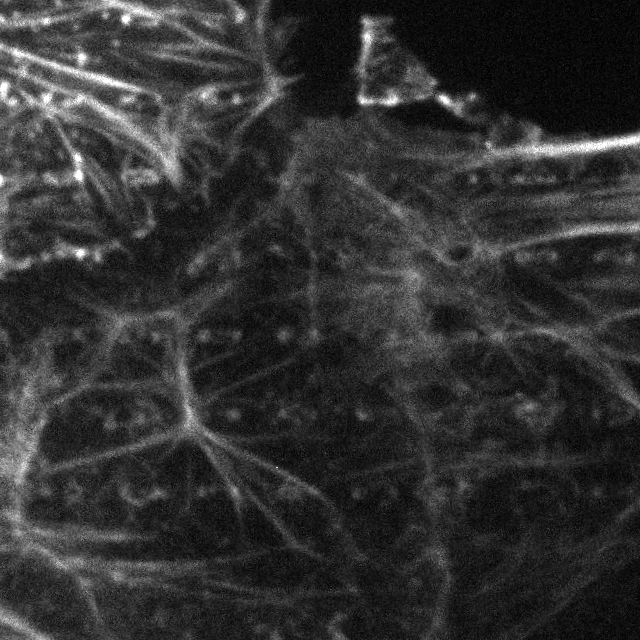

Supplement: Supplementary file 11 — Source data Fig. 3 [file 44318_2025_677_MOESM11_ESM.zip › Figure 3/3E/U2OS cell Images/20uM ML141_74_w2CSU GFP_640x640.tif]

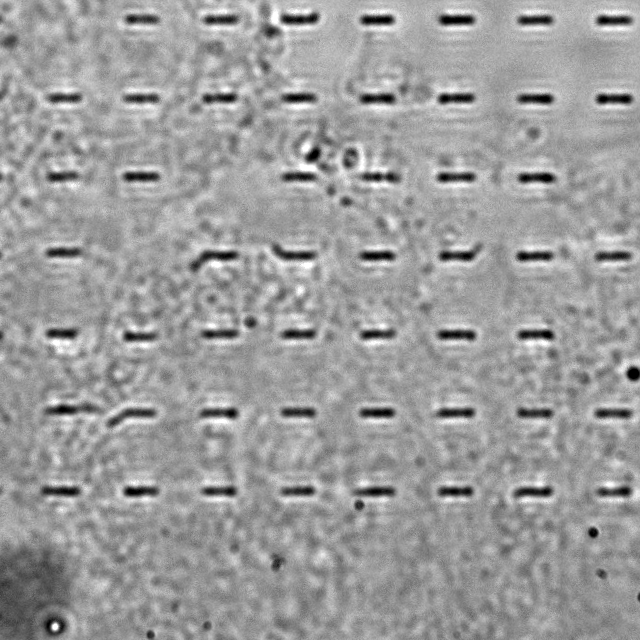

Supplement: Supplementary file 11 — Source data Fig. 3 [file 44318_2025_677_MOESM11_ESM.zip › Figure 3/3E/U2OS cell Images/20uM ML141_74_w3Trans_640x640.tif]

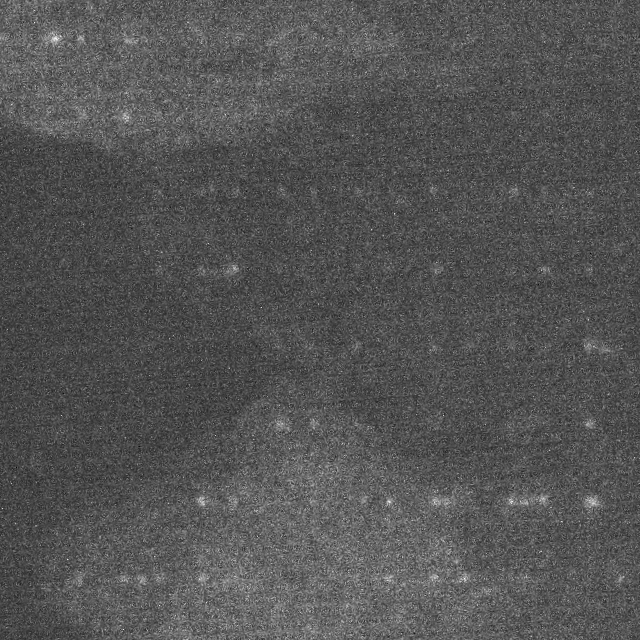

Supplement: Supplementary file 11 — Source data Fig. 3 [file 44318_2025_677_MOESM11_ESM.zip › Figure 3/3E/U2OS cell Images/50uM ML141_NANOBAR_13_w1CSU mCherry_640x640.tif]

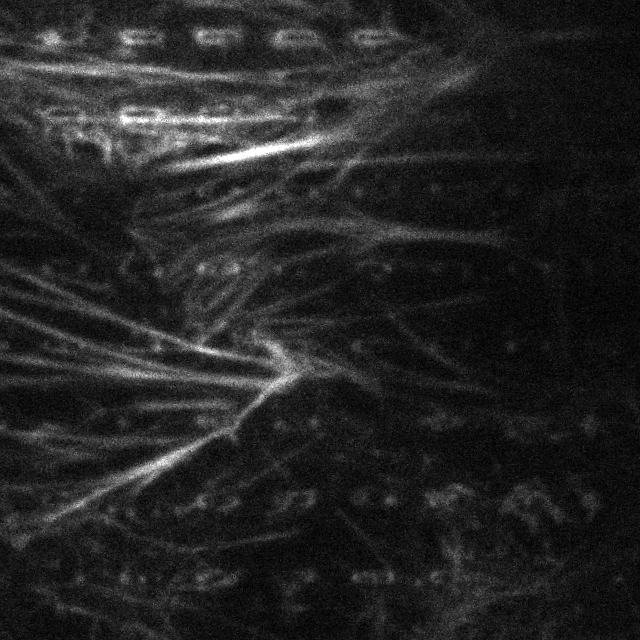

Supplement: Supplementary file 11 — Source data Fig. 3 [file 44318_2025_677_MOESM11_ESM.zip › Figure 3/3E/U2OS cell Images/50uM ML141_NANOBAR_13_w2CSU GFP_640x640.tif]

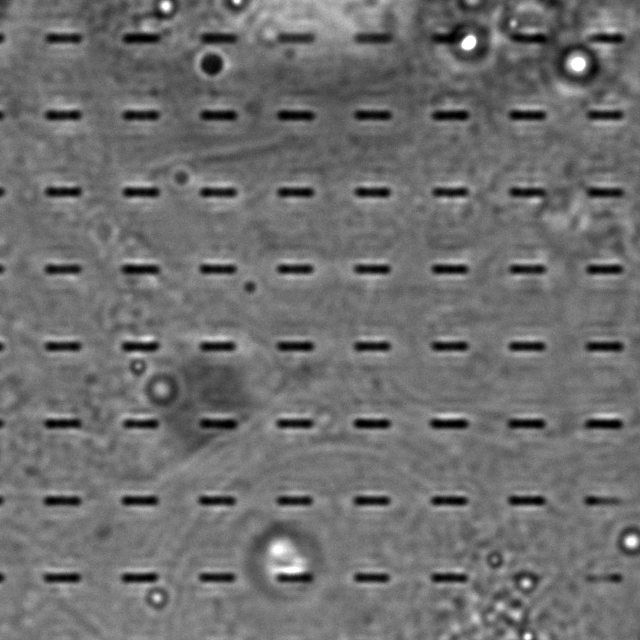

Supplement: Supplementary file 11 — Source data Fig. 3 [file 44318_2025_677_MOESM11_ESM.zip › Figure 3/3E/U2OS cell Images/50uM ML141_NANOBAR_13_w3Trans_640x640.tif]

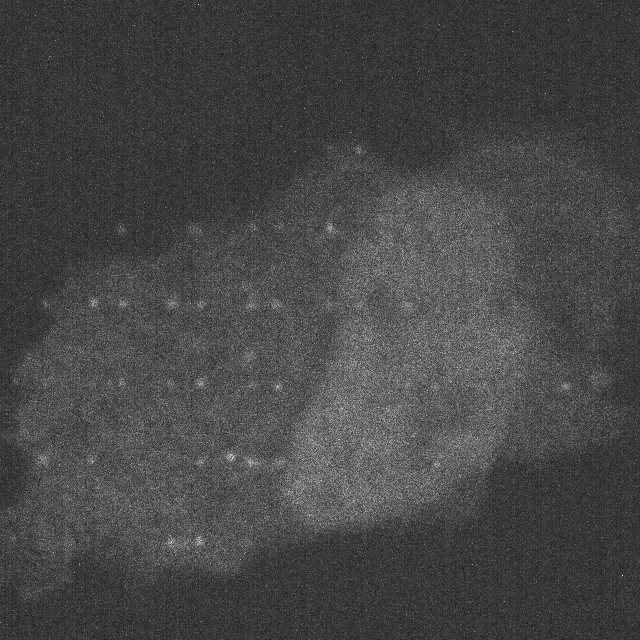

Supplement: Supplementary file 11 — Source data Fig. 3 [file 44318_2025_677_MOESM11_ESM.zip › Figure 3/3E/U2OS cell Images/DMSO_NANOBAR_3_w1CSU mCherry_640x640.tif]

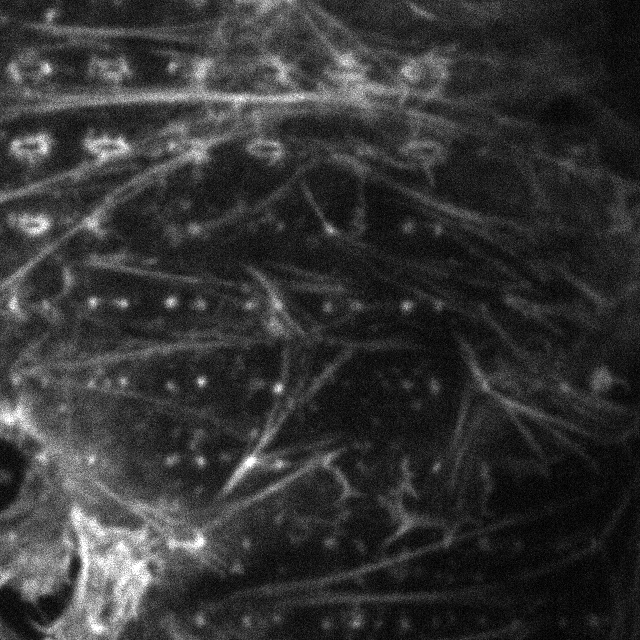

Supplement: Supplementary file 11 — Source data Fig. 3 [file 44318_2025_677_MOESM11_ESM.zip › Figure 3/3E/U2OS cell Images/DMSO_NANOBAR_3_w2CSU GFP_640x640.tif]

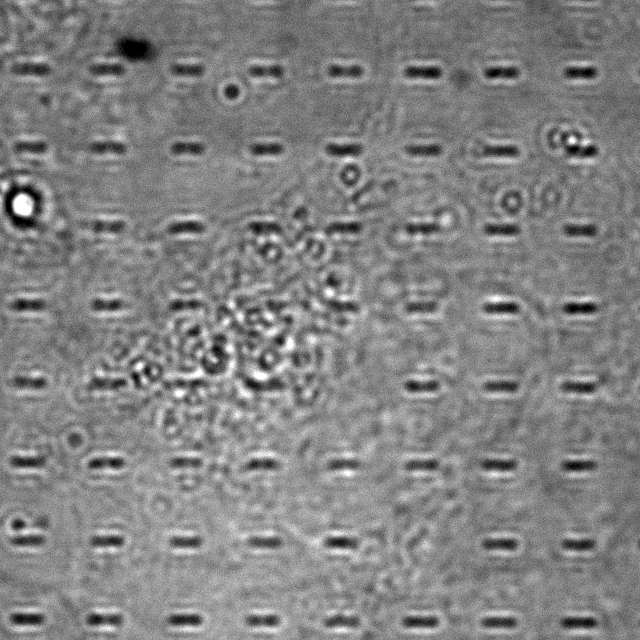

Supplement: Supplementary file 11 — Source data Fig. 3 [file 44318_2025_677_MOESM11_ESM.zip › Figure 3/3E/U2OS cell Images/DMSO_NANOBAR_3_w3Trans_640x640.tif]

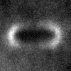

Supplement: Supplementary file 11 — Source data Fig. 3 [file 44318_2025_677_MOESM11_ESM.zip › Figure 3/3H/3H_20uM ML141_anti-FBP17_16bit_stack.tif]

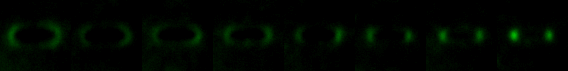

Supplement: Supplementary file 11 — Source data Fig. 3 [file 44318_2025_677_MOESM11_ESM.zip › Figure 3/3H/3H_20uM ML141_anti-FBP17_Montage.tif]

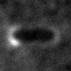

Supplement: Supplementary file 11 — Source data Fig. 3 [file 44318_2025_677_MOESM11_ESM.zip › Figure 3/3H/3H_50uM ML141_anti-FBP17_16bit_stack.tif]

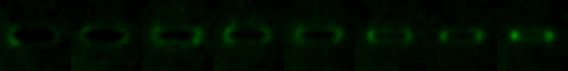

Supplement: Supplementary file 11 — Source data Fig. 3 [file 44318_2025_677_MOESM11_ESM.zip › Figure 3/3H/3H_50uM ML141_anti-FBP17_Montage.tif]

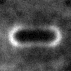

Supplement: Supplementary file 11 — Source data Fig. 3 [file 44318_2025_677_MOESM11_ESM.zip › Figure 3/3H/3H_DMSO_anti-FBP17_16bit_stack.tif]

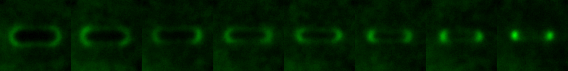

Supplement: Supplementary file 11 — Source data Fig. 3 [file 44318_2025_677_MOESM11_ESM.zip › Figure 3/3H/3H_DMSO_anti-FBP17_Montage.tif]

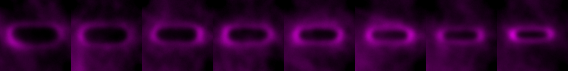

Supplement: Supplementary file 11 — Source data Fig. 3 [file 44318_2025_677_MOESM11_ESM.zip › Figure 3/3J/3J_20uM ML141_Phalloidin-565.tif]

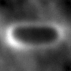

Supplement: Supplementary file 11 — Source data Fig. 3 [file 44318_2025_677_MOESM11_ESM.zip › Figure 3/3J/3J_20uM ML141_Phalloidin-565_16bit_stack.tif]

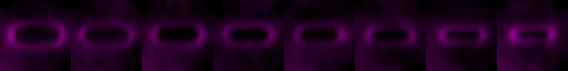

Supplement: Supplementary file 11 — Source data Fig. 3 [file 44318_2025_677_MOESM11_ESM.zip › Figure 3/3J/3J_50uM ML141_Phalloidin-565.tif]

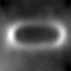

Supplement: Supplementary file 11 — Source data Fig. 3 [file 44318_2025_677_MOESM11_ESM.zip › Figure 3/3J/3J_50uM ML141_Phalloidin-565_16bit_stack.tif]

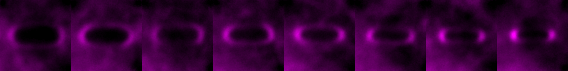

Supplement: Supplementary file 11 — Source data Fig. 3 [file 44318_2025_677_MOESM11_ESM.zip › Figure 3/3J/3J_DMSO_Phalloidin-565.tif]

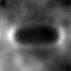

Supplement: Supplementary file 11 — Source data Fig. 3 [file 44318_2025_677_MOESM11_ESM.zip › Figure 3/3J/3J_DMSO_Phalloidin-565_16bit_stack.tif]

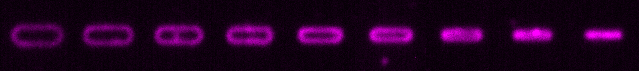

Supplement: Supplementary file 12 — Source data Fig. 4 [file 44318_2025_677_MOESM12_ESM.zip › Figure 4/4C/F+C.tif]

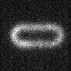

Supplement: Supplementary file 12 — Source data Fig. 4 [file 44318_2025_677_MOESM12_ESM.zip › Figure 4/4C/F+C_16bit_stack.tif]

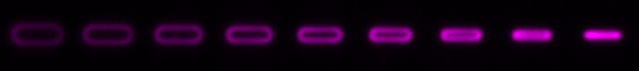

Supplement: Supplementary file 12 — Source data Fig. 4 [file 44318_2025_677_MOESM12_ESM.zip › Figure 4/4C/F.tif]

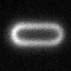

Supplement: Supplementary file 12 — Source data Fig. 4 [file 44318_2025_677_MOESM12_ESM.zip › Figure 4/4C/F_16bit_stack.tif]

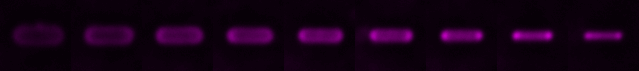

Supplement: Supplementary file 12 — Source data Fig. 4 [file 44318_2025_677_MOESM12_ESM.zip › Figure 4/4F/dHR1+C.tif]
