# Supplementary figures and images for: Membrane curvature initiates Cdc42-FBP17-N-WASP clustering and actin nucleation (part 2 of 2)
Source: EMBO J. 2026 Jan 3;45(3):953–86. doi: 10.1038/s44318-025-00677-w (PMC12864879; doi:10.1038/s44318-025-00677-w)

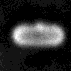

Supplement: Supplementary file 12 — Source data Fig. 4 [file 44318_2025_677_MOESM12_ESM.zip › Figure 4/4F/dHR1+C_16bit_stack.tif]

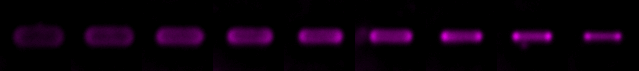

Supplement: Supplementary file 12 — Source data Fig. 4 [file 44318_2025_677_MOESM12_ESM.zip › Figure 4/4F/dHR1.tif]

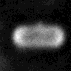

Supplement: Supplementary file 12 — Source data Fig. 4 [file 44318_2025_677_MOESM12_ESM.zip › Figure 4/4F/dHR1_16bit_stack.tif]

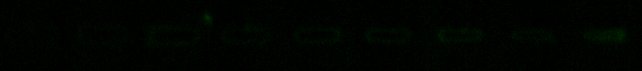

Supplement: Supplementary file 12 — Source data Fig. 4 [file 44318_2025_677_MOESM12_ESM.zip › Figure 4/4I/Figure 4I_0s.tif]

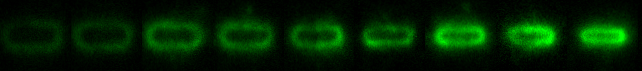

Supplement: Supplementary file 12 — Source data Fig. 4 [file 44318_2025_677_MOESM12_ESM.zip › Figure 4/4I/Figure 4I_180s.tif]

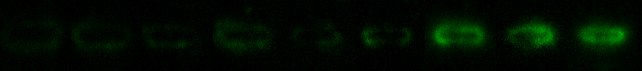

Supplement: Supplementary file 12 — Source data Fig. 4 [file 44318_2025_677_MOESM12_ESM.zip › Figure 4/4I/Figure 4I_90s.tif]

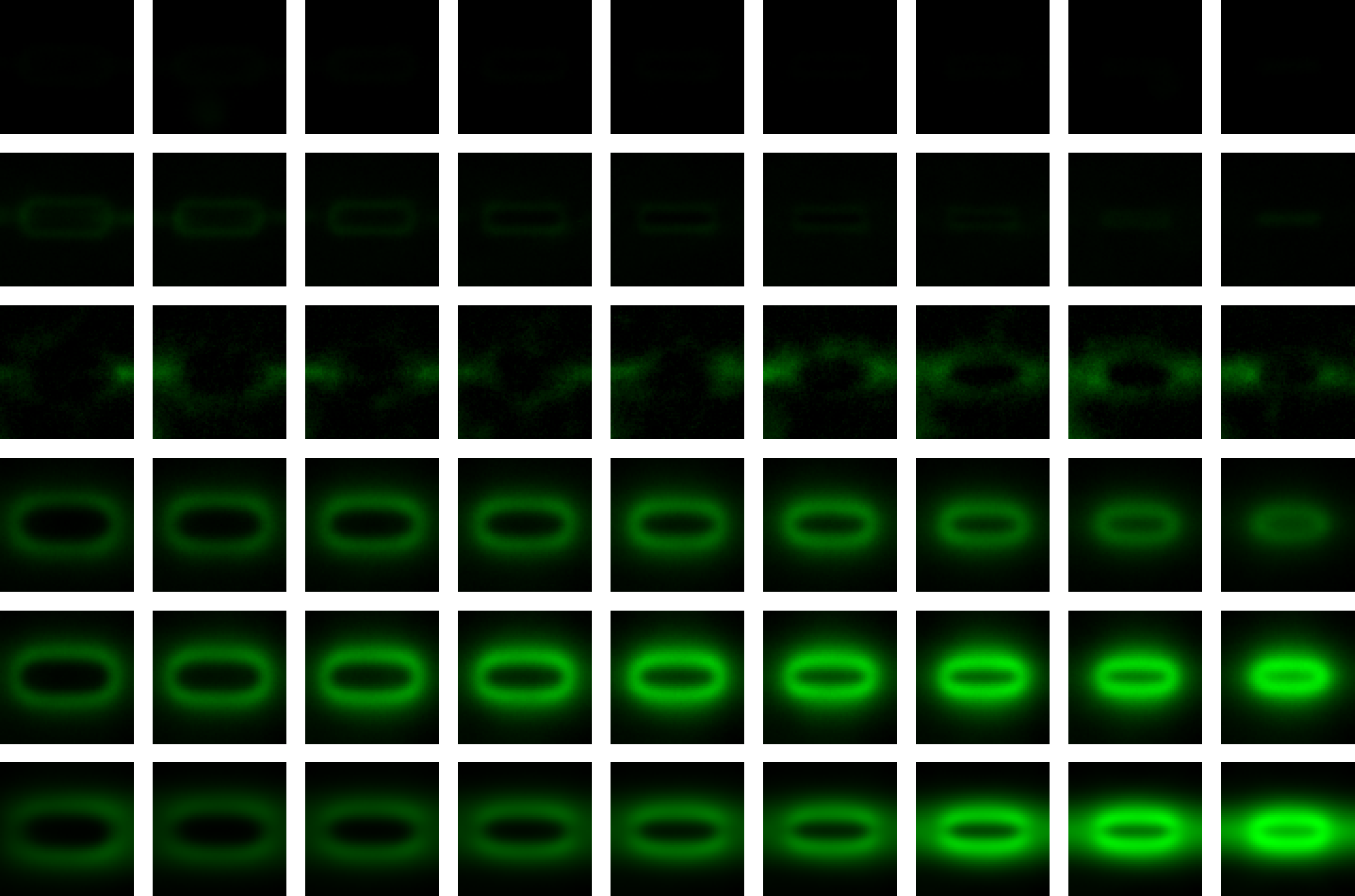

Supplement: Supplementary file 12 — Source data Fig. 4 [file 44318_2025_677_MOESM12_ESM.zip › Figure 4/4L/4L_Actin on nanobar.tif]

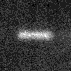

Supplement: Supplementary file 12 — Source data Fig. 4 [file 44318_2025_677_MOESM12_ESM.zip › Figure 4/4L/4L_F+A+C_stack.tif]

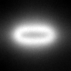

Supplement: Supplementary file 12 — Source data Fig. 4 [file 44318_2025_677_MOESM12_ESM.zip › Figure 4/4L/4L_F+N+A+C_stack.tif]

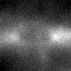

Supplement: Supplementary file 12 — Source data Fig. 4 [file 44318_2025_677_MOESM12_ESM.zip › Figure 4/4L/4L_F+N+A_stack.tif]

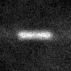

Supplement: Supplementary file 12 — Source data Fig. 4 [file 44318_2025_677_MOESM12_ESM.zip › Figure 4/4L/4L_F+N+C_stack.tif]

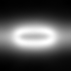

Supplement: Supplementary file 12 — Source data Fig. 4 [file 44318_2025_677_MOESM12_ESM.zip › Figure 4/4L/4L_FdHR1+N+A+C_stack.tif]

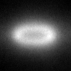

Supplement: Supplementary file 12 — Source data Fig. 4 [file 44318_2025_677_MOESM12_ESM.zip › Figure 4/4L/4L_N+A+C_stack.tif]

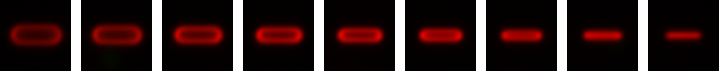

Supplement: Supplementary file 12 — Source data Fig. 4 [file 44318_2025_677_MOESM12_ESM.zip › Figure 4/4L/4L_SLB on nanobar.tiff]

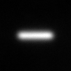

Supplement: Supplementary file 12 — Source data Fig. 4 [file 44318_2025_677_MOESM12_ESM.zip › Figure 4/4L/4L_SLB_STACK.tif]

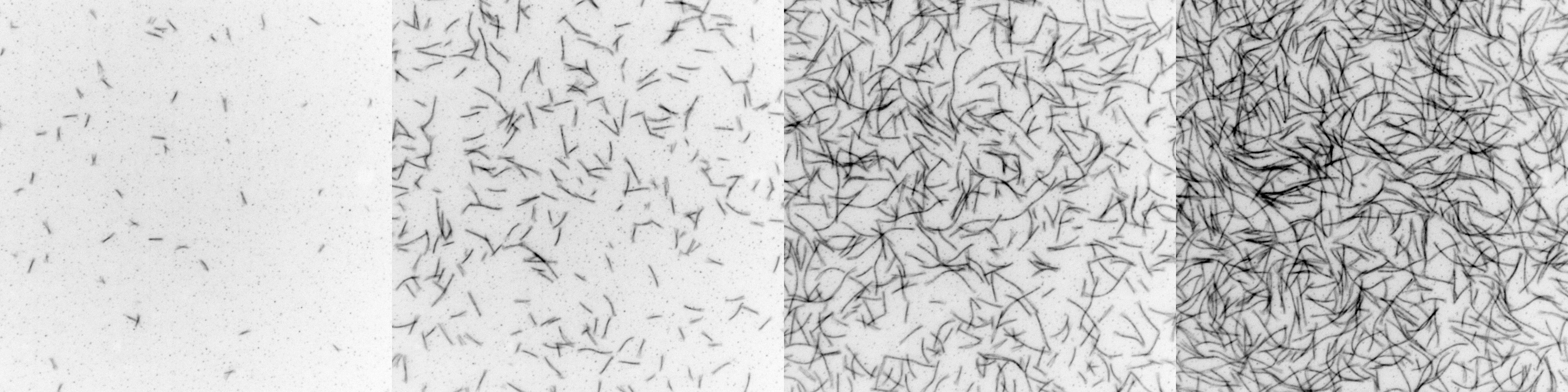

Supplement: Supplementary file 13 — Source data Fig. 5 [file 44318_2025_677_MOESM13_ESM.zip › Figure 5/5B/5B_Actin only_16bit.tif]

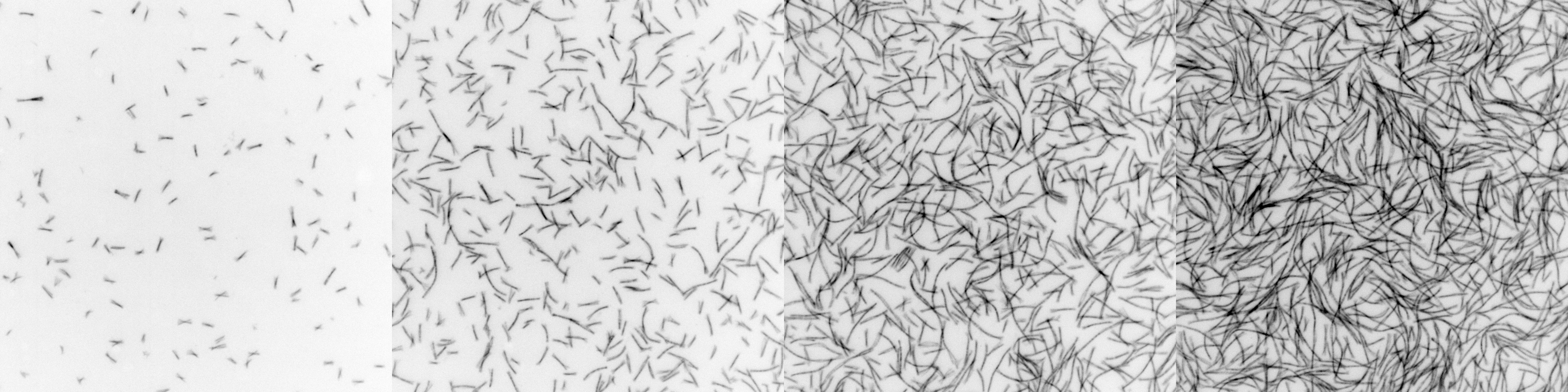

Supplement: Supplementary file 13 — Source data Fig. 5 [file 44318_2025_677_MOESM13_ESM.zip › Figure 5/5B/5B_F+A_16bit.tif]

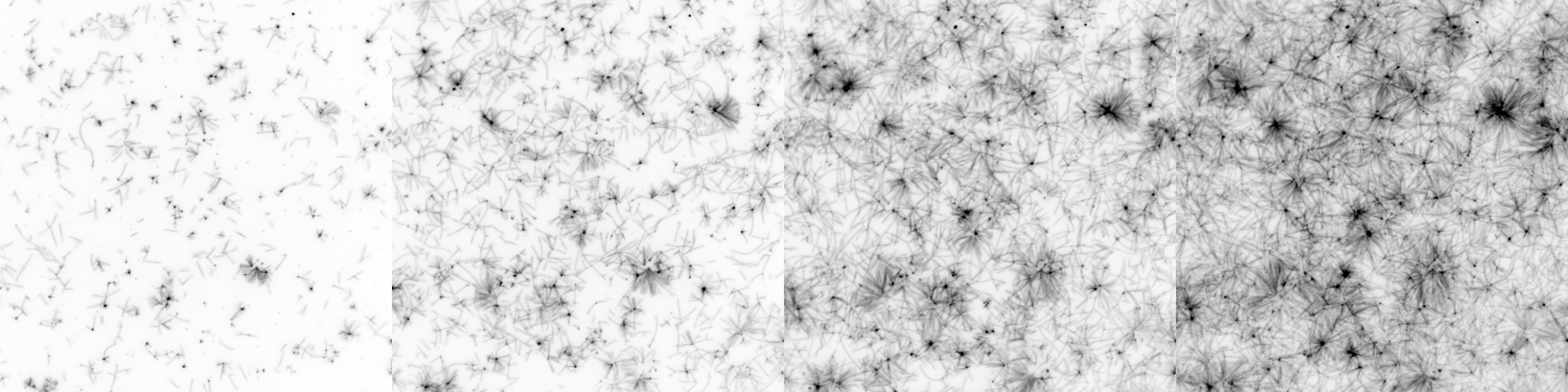

Supplement: Supplementary file 13 — Source data Fig. 5 [file 44318_2025_677_MOESM13_ESM.zip › Figure 5/5B/5B_F+N+A+C_16bit.tif]

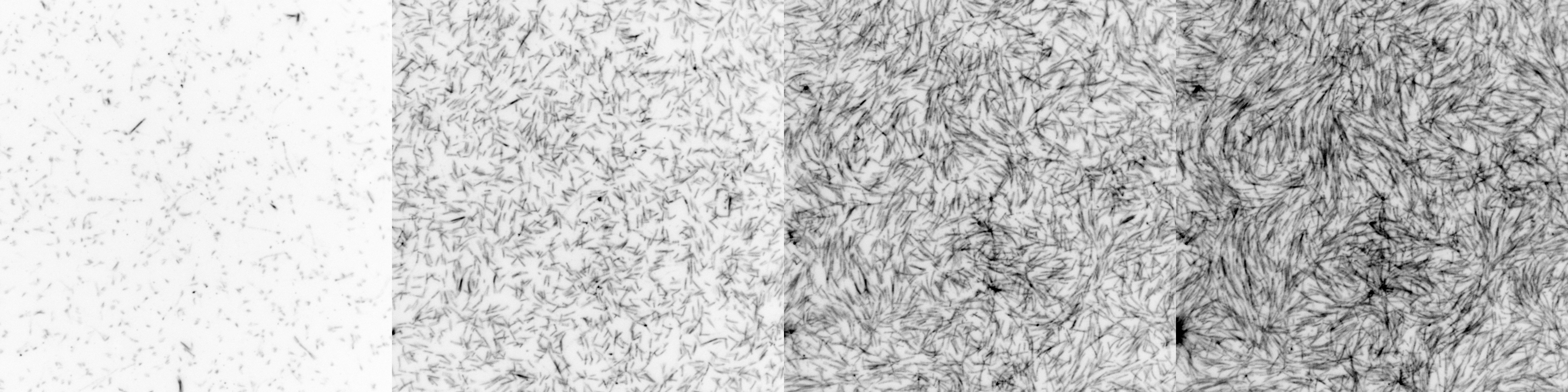

Supplement: Supplementary file 13 — Source data Fig. 5 [file 44318_2025_677_MOESM13_ESM.zip › Figure 5/5B/5B_F+N+A_16bit.tif]

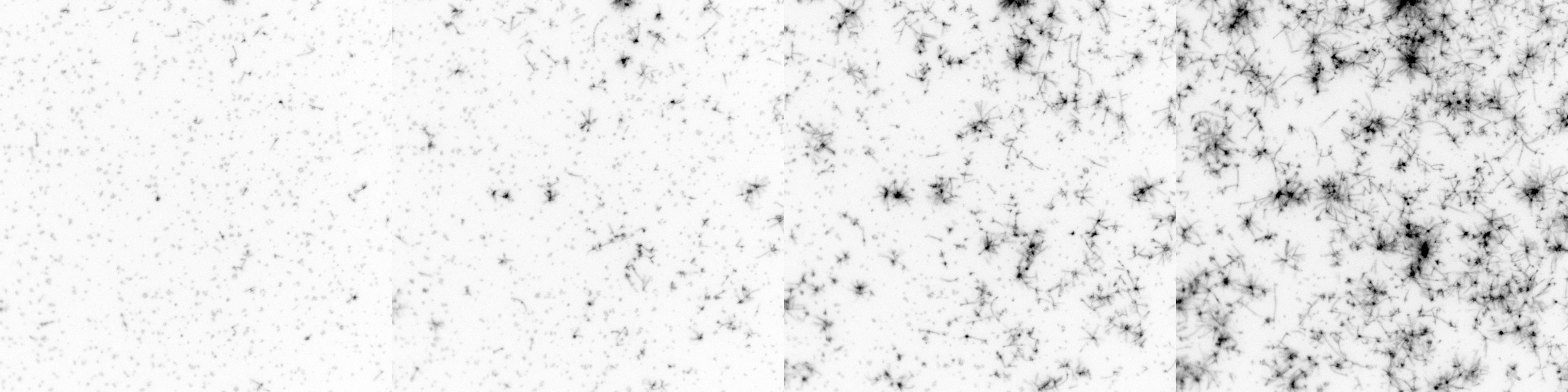

Supplement: Supplementary file 13 — Source data Fig. 5 [file 44318_2025_677_MOESM13_ESM.zip › Figure 5/5B/5B_FdHR1+N+A+C_16bit.tif]

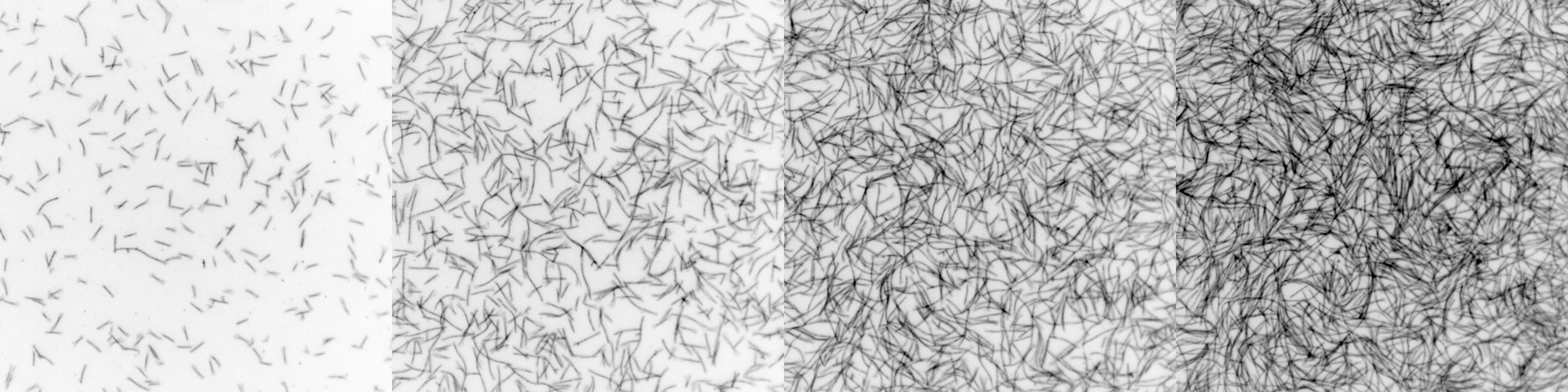

Supplement: Supplementary file 13 — Source data Fig. 5 [file 44318_2025_677_MOESM13_ESM.zip › Figure 5/5B/5B_N+A+C_16bit.tif]

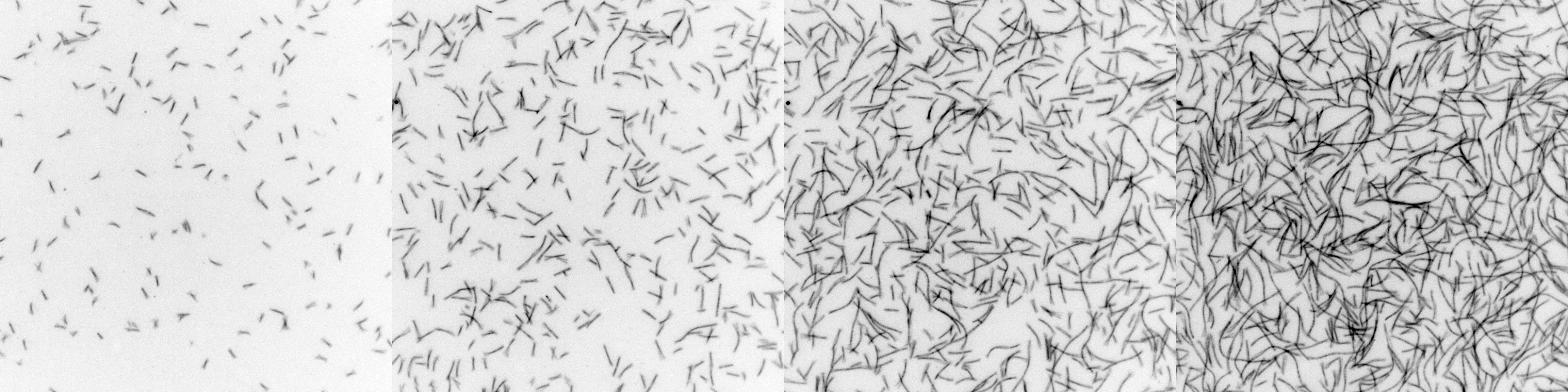

Supplement: Supplementary file 13 — Source data Fig. 5 [file 44318_2025_677_MOESM13_ESM.zip › Figure 5/5B/5B_N+A_16bit.tif]

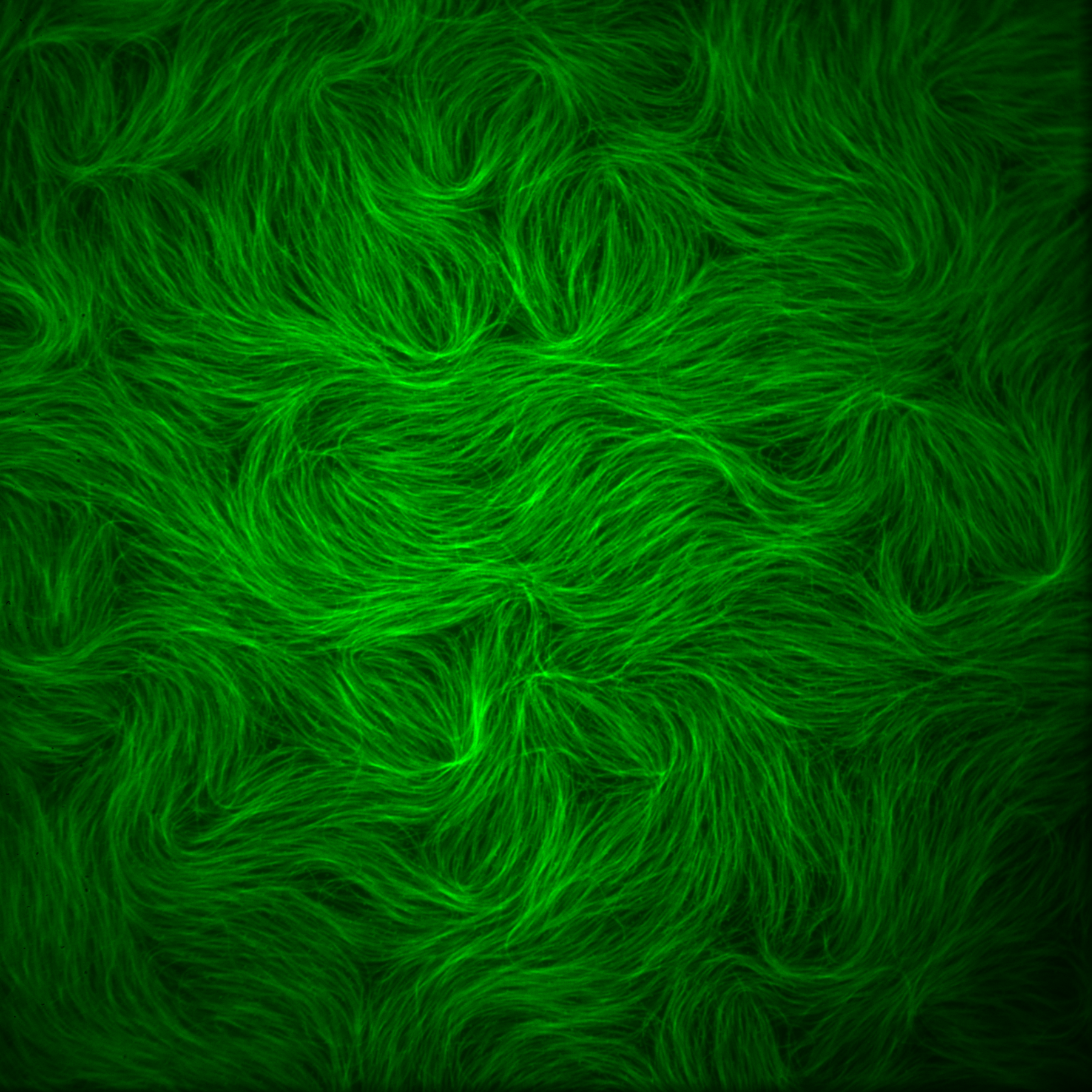

Supplement: Supplementary file 13 — Source data Fig. 5 [file 44318_2025_677_MOESM13_ESM.zip › Figure 5/5E/5E_Actin only.tif]

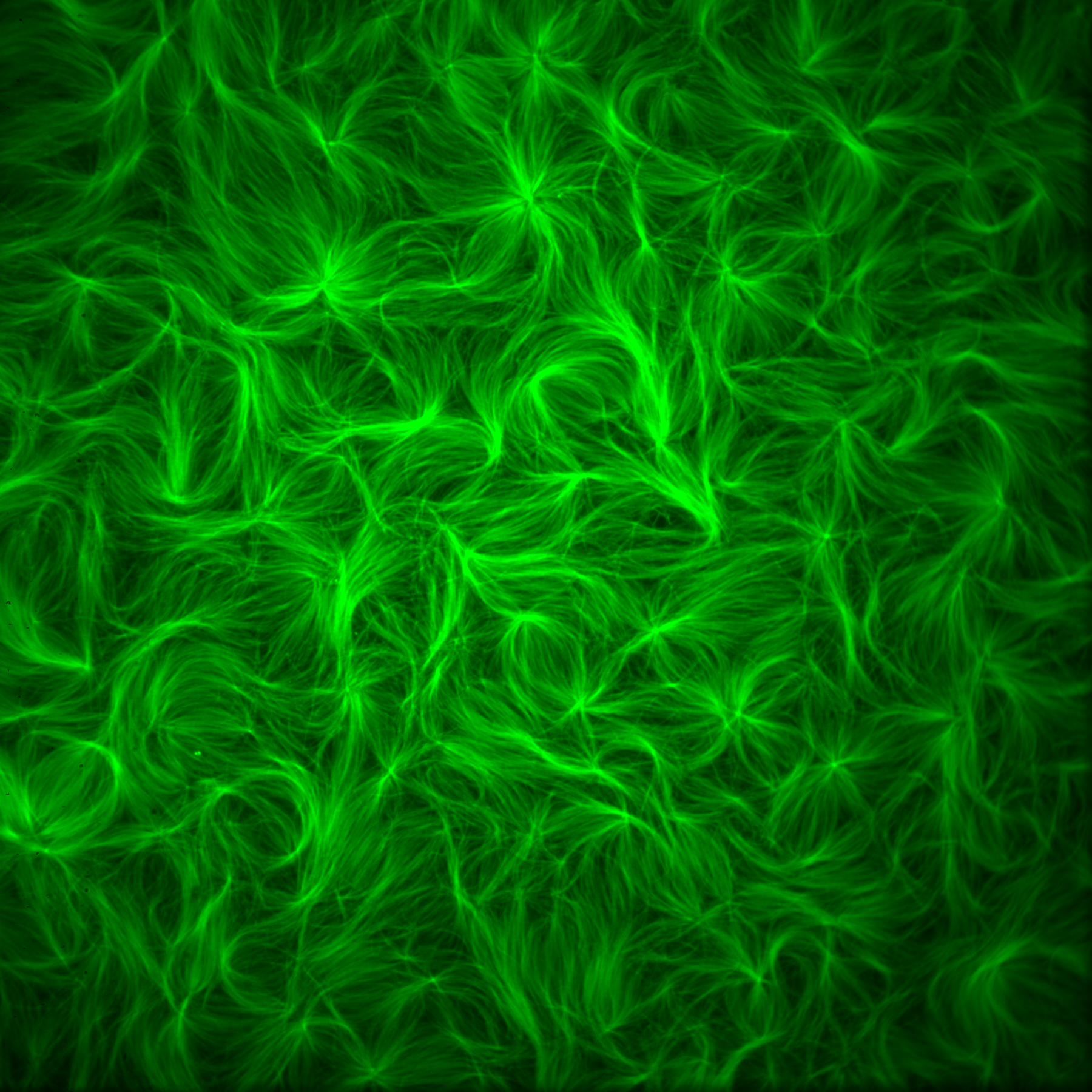

Supplement: Supplementary file 13 — Source data Fig. 5 [file 44318_2025_677_MOESM13_ESM.zip › Figure 5/5E/5E_F+A.tif]

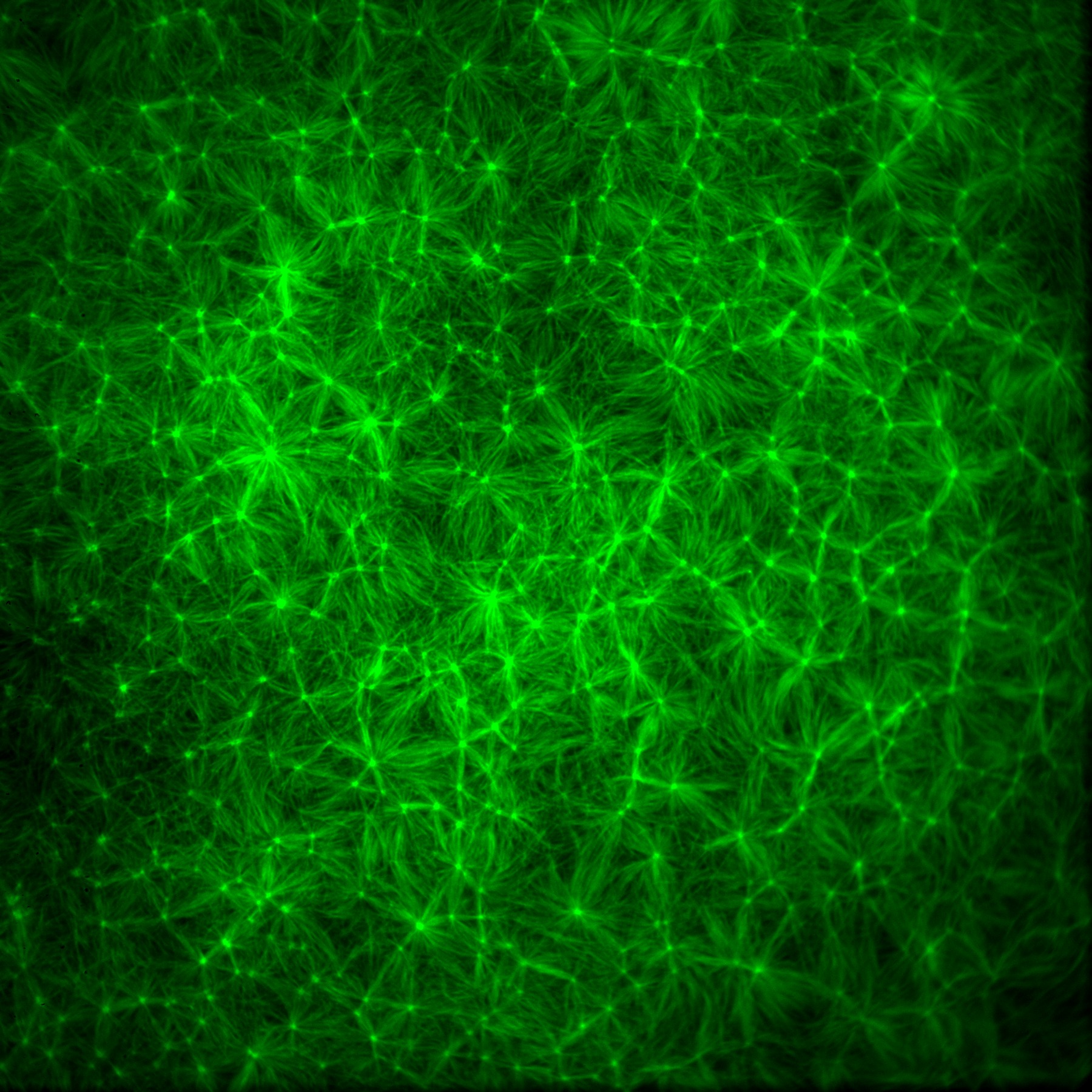

Supplement: Supplementary file 13 — Source data Fig. 5 [file 44318_2025_677_MOESM13_ESM.zip › Figure 5/5E/5E_F+N+A+C.tif]

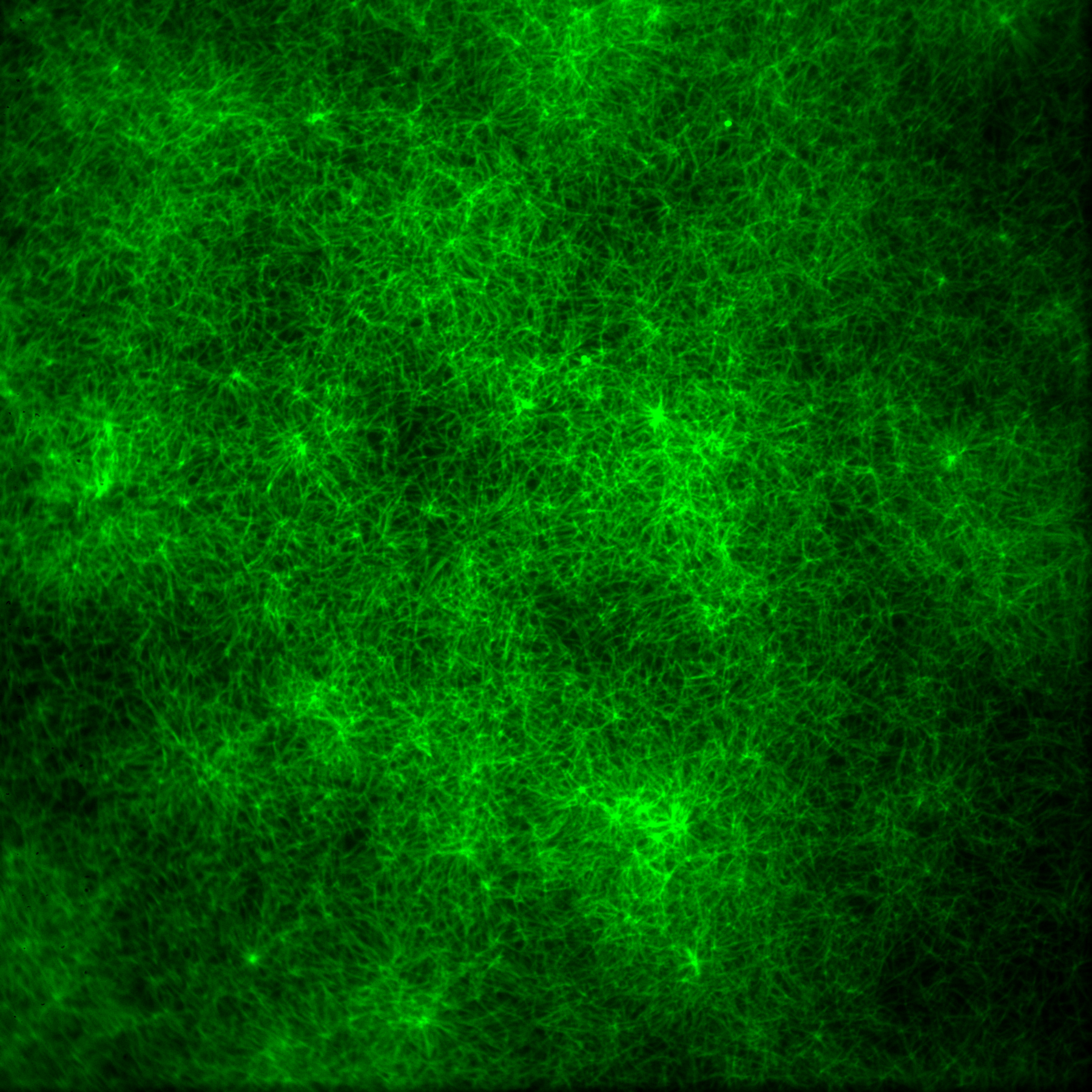

Supplement: Supplementary file 13 — Source data Fig. 5 [file 44318_2025_677_MOESM13_ESM.zip › Figure 5/5E/5E_F+N+A.tif]

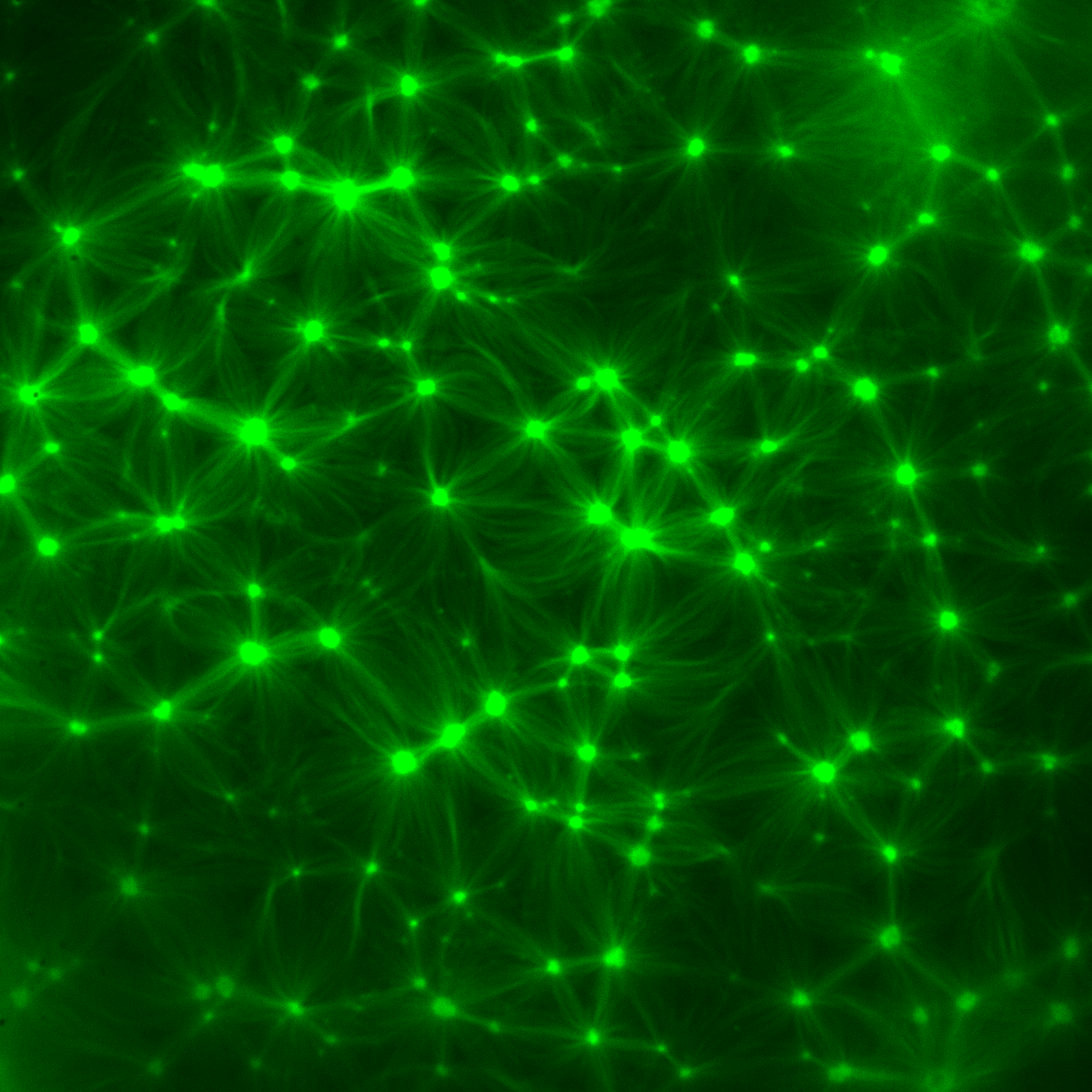

Supplement: Supplementary file 13 — Source data Fig. 5 [file 44318_2025_677_MOESM13_ESM.zip › Figure 5/5E/5E_FdHR1+N+A+C.tif]

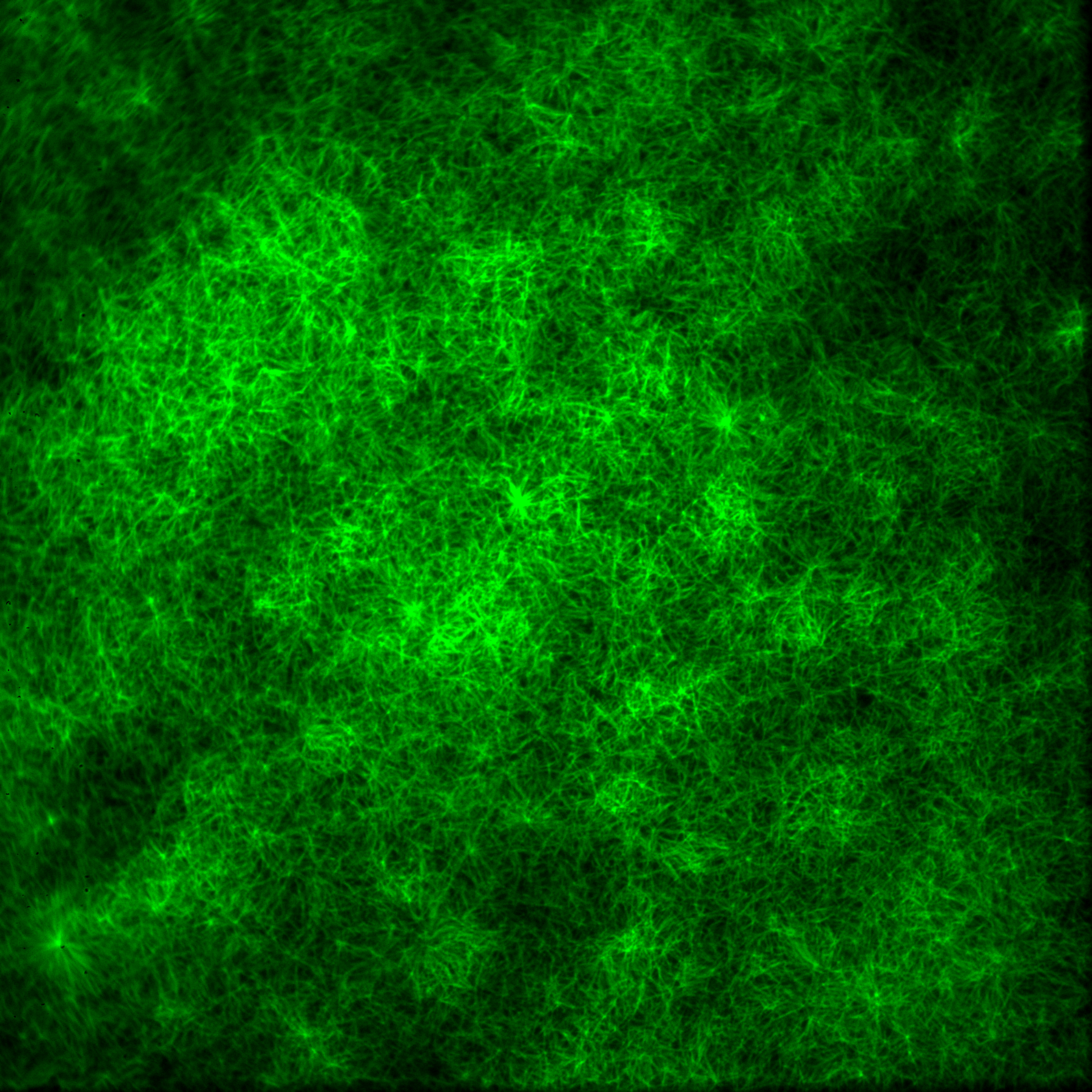

Supplement: Supplementary file 13 — Source data Fig. 5 [file 44318_2025_677_MOESM13_ESM.zip › Figure 5/5E/5E_N+A+C.tif]

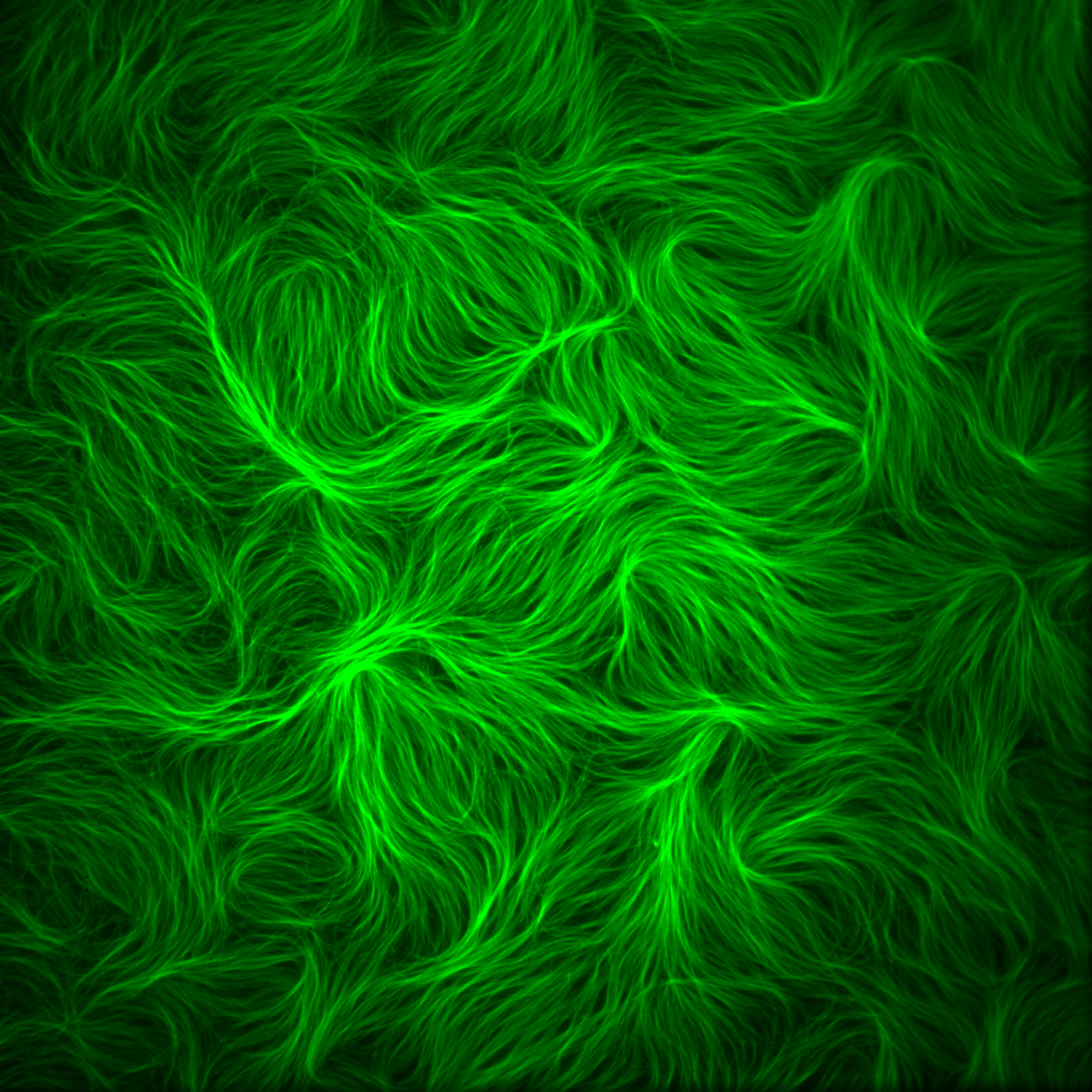

Supplement: Supplementary file 13 — Source data Fig. 5 [file 44318_2025_677_MOESM13_ESM.zip › Figure 5/5E/5E_N+A.tif]

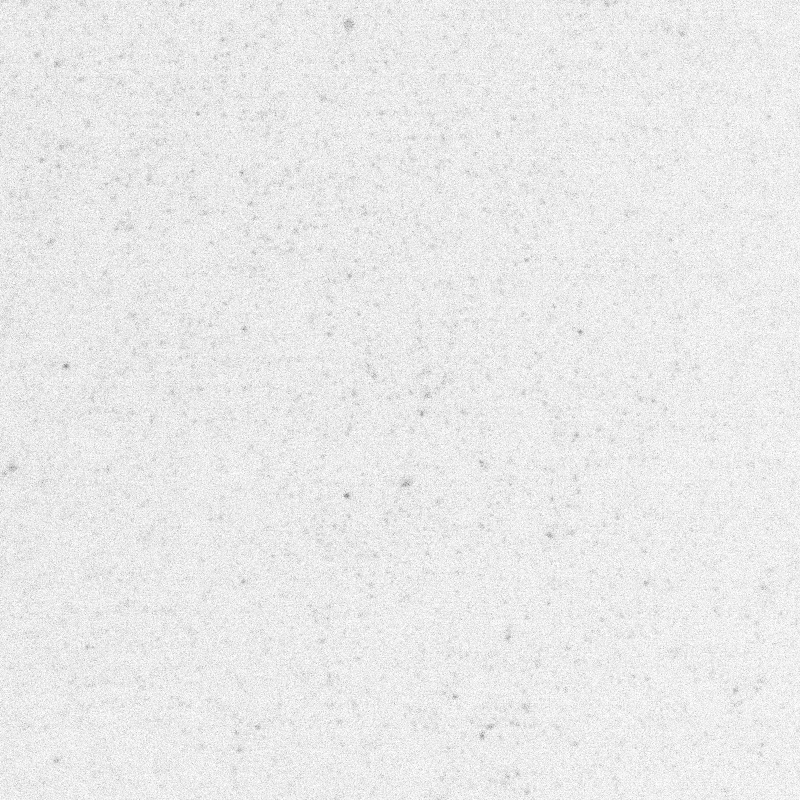

Supplement: Supplementary file 14 — Source data Fig. 6 [file 44318_2025_677_MOESM14_ESM.zip › Figure 6/6H/6H_FBP17+N-WASP+Arp23+CapZ_stack.tif]

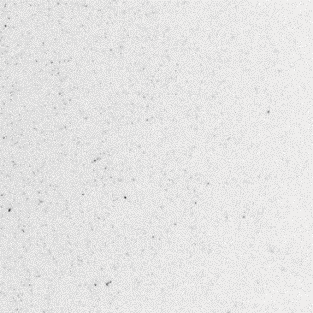

Supplement: Supplementary file 14 — Source data Fig. 6 [file 44318_2025_677_MOESM14_ESM.zip › Figure 6/6H/6H_FBP17+N-WASP+Arp23_stack.tif]

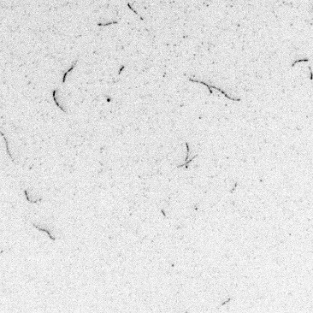

Supplement: Supplementary file 14 — Source data Fig. 6 [file 44318_2025_677_MOESM14_ESM.zip › Figure 6/6H/6H_N-WASP+Arp23_stack.tif]

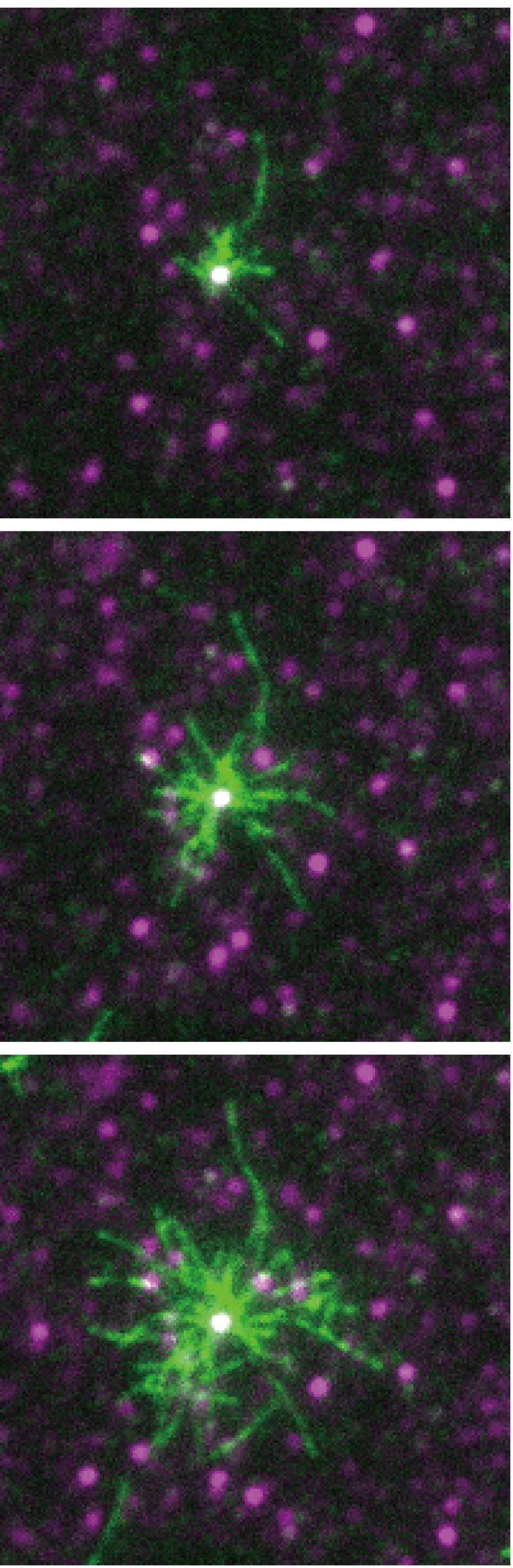

Supplement: Supplementary file 14 — Source data Fig. 6 [file 44318_2025_677_MOESM14_ESM.zip › Figure 6/6J/6J_Dual color TIRF actin image.tif]

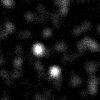

Supplement: Supplementary file 15 — Source data Fig. 7 [file 44318_2025_677_MOESM15_ESM.zip › Figure 7/7B/7B_FBP17 clusters withN-WASP_16bit.tif]

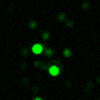

Supplement: Supplementary file 15 — Source data Fig. 7 [file 44318_2025_677_MOESM15_ESM.zip › Figure 7/7B/7B_N-WASP clusters withFBP17.tif]

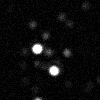

Supplement: Supplementary file 15 — Source data Fig. 7 [file 44318_2025_677_MOESM15_ESM.zip › Figure 7/7B/7B_N-WASP clusters withFBP17_16bit.tif]

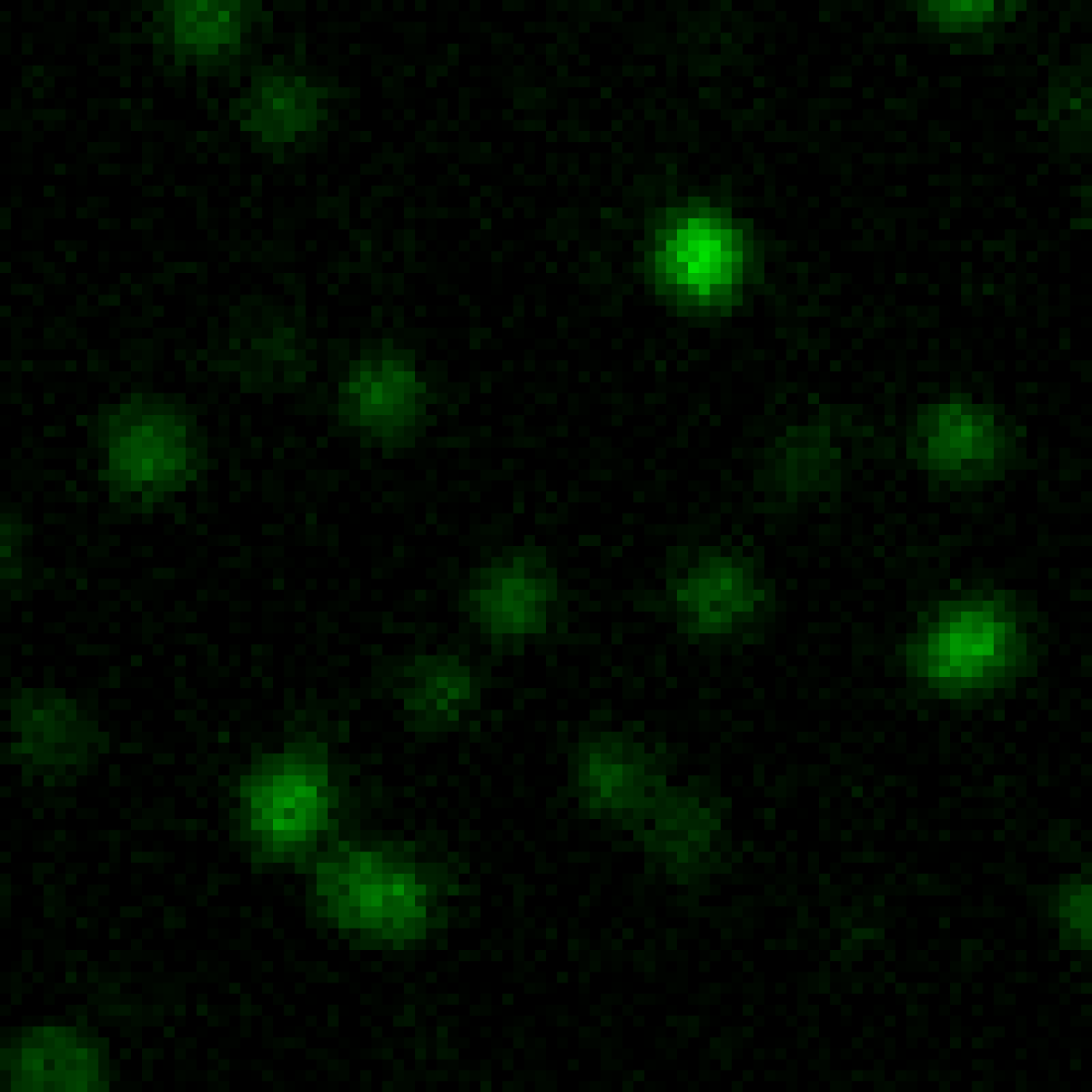

Supplement: Supplementary file 15 — Source data Fig. 7 [file 44318_2025_677_MOESM15_ESM.zip › Figure 7/7B/7B_N-WASP FL control.tif]

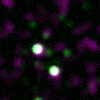

Supplement: Supplementary file 15 — Source data Fig. 7 [file 44318_2025_677_MOESM15_ESM.zip › Figure 7/7B/7B_N-WASP+FBP17 clusters merge.tif]

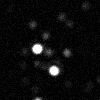

Supplement: Supplementary file 15 — Source data Fig. 7 [file 44318_2025_677_MOESM15_ESM.zip › Figure 7/7B/7B_N-WASP+FBP17 clusters merge_16bit.tif]

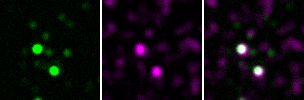

Supplement: Supplementary file 15 — Source data Fig. 7 [file 44318_2025_677_MOESM15_ESM.zip › Figure 7/7B/7B_N-WASP+FBP17 single particle.tiff]

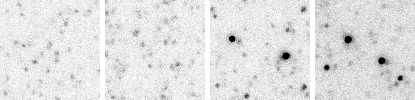

Supplement: Supplementary file 15 — Source data Fig. 7 [file 44318_2025_677_MOESM15_ESM.zip › Figure 7/7F/Figure 7F.tif]

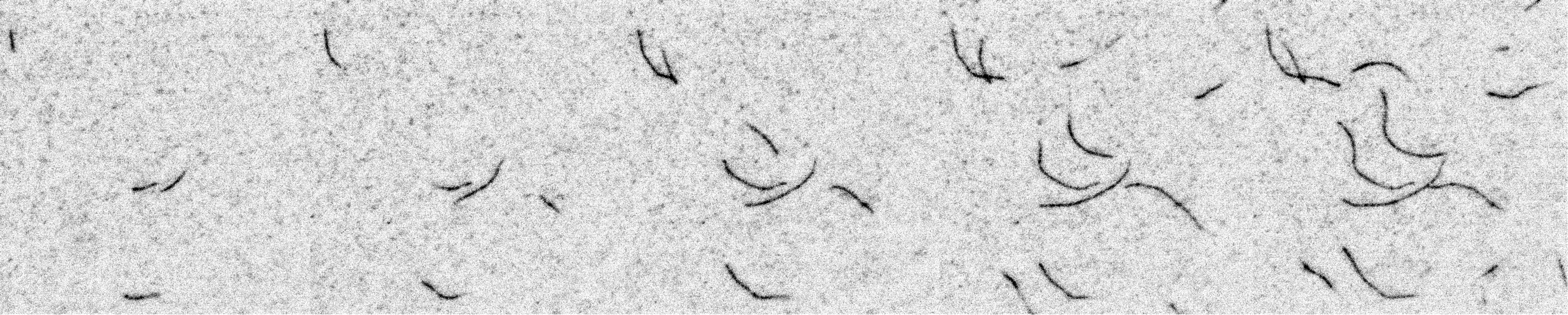

Supplement: Supplementary file 15 — Source data Fig. 7 [file 44318_2025_677_MOESM15_ESM.zip › Figure 7/7H/7H_+SH3 mono.tif]

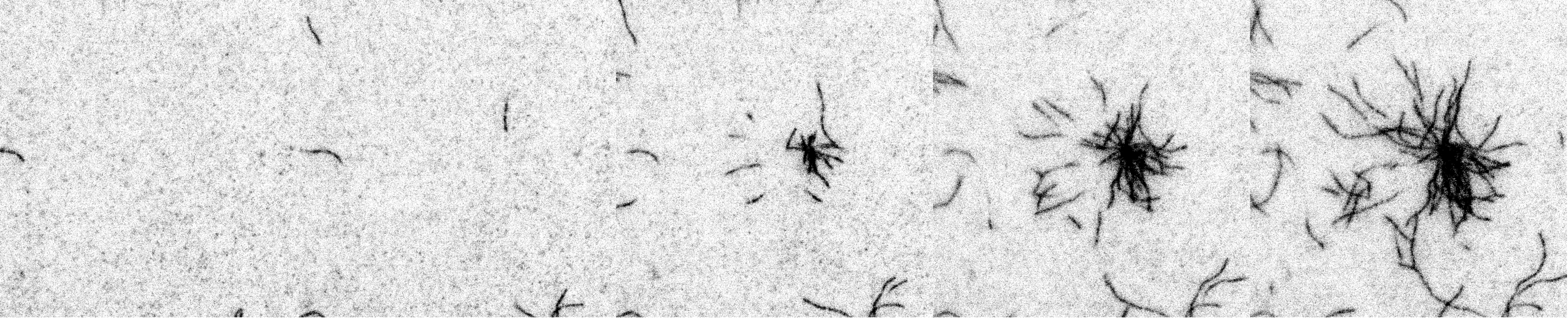

Supplement: Supplementary file 15 — Source data Fig. 7 [file 44318_2025_677_MOESM15_ESM.zip › Figure 7/7H/7H_+SH3 tetramer.tif]

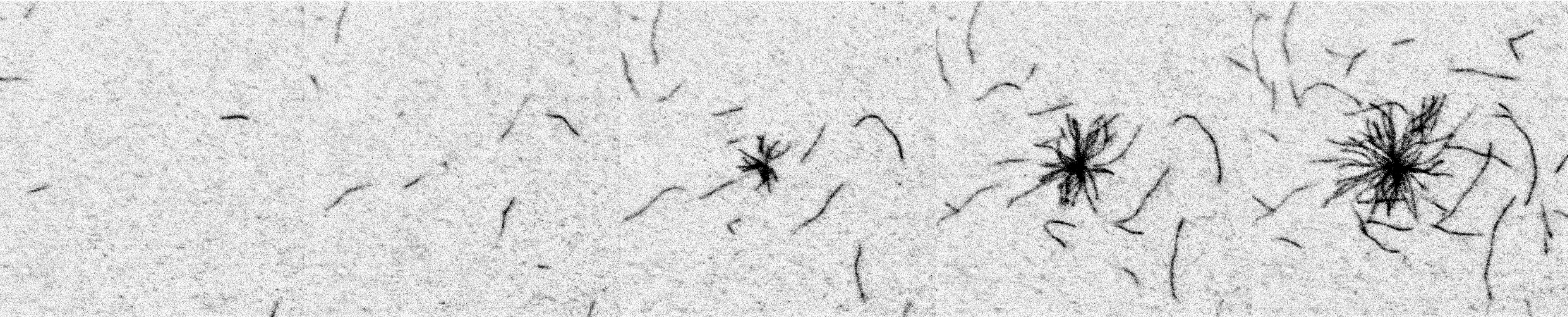

Supplement: Supplementary file 15 — Source data Fig. 7 [file 44318_2025_677_MOESM15_ESM.zip › Figure 7/7H/7H_+SH3 trimer.tif]

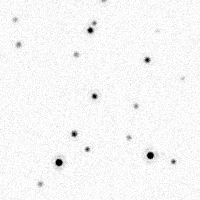

Supplement: Supplementary file 16 — Source data Fig. 8 [file 44318_2025_677_MOESM16_ESM.zip › Figure 8/8C/8C_FBP17_Single particle.tif]

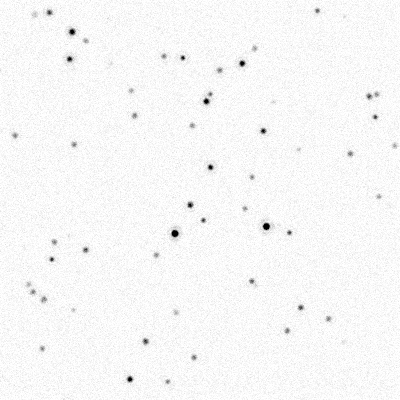

Supplement: Supplementary file 16 — Source data Fig. 8 [file 44318_2025_677_MOESM16_ESM.zip › Figure 8/8C/8C_FBP17_Single particle_Full view.tif]

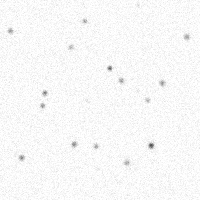

Supplement: Supplementary file 16 — Source data Fig. 8 [file 44318_2025_677_MOESM16_ESM.zip › Figure 8/8C/8C_K166A_Single particle.tif]

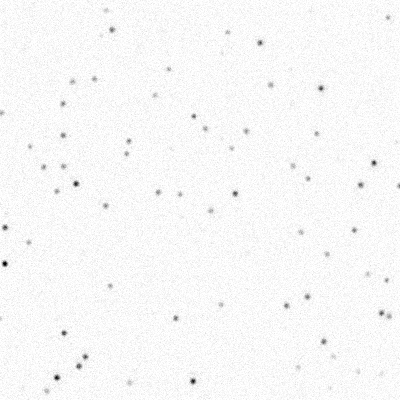

Supplement: Supplementary file 16 — Source data Fig. 8 [file 44318_2025_677_MOESM16_ESM.zip › Figure 8/8C/8C_K166A_Single particle_Full view.tif]

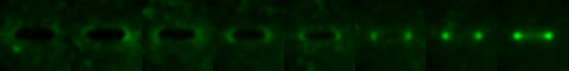

Supplement: Supplementary file 16 — Source data Fig. 8 [file 44318_2025_677_MOESM16_ESM.zip › Figure 8/8F/8F_FBP17.tiff]

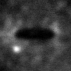

Supplement: Supplementary file 16 — Source data Fig. 8 [file 44318_2025_677_MOESM16_ESM.zip › Figure 8/8F/8F_FBP17_16bit_stack.tif]

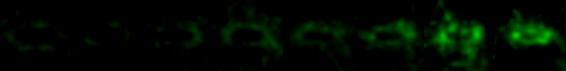

Supplement: Supplementary file 16 — Source data Fig. 8 [file 44318_2025_677_MOESM16_ESM.zip › Figure 8/8F/8F_FdHR1.tiff]

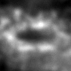

Supplement: Supplementary file 16 — Source data Fig. 8 [file 44318_2025_677_MOESM16_ESM.zip › Figure 8/8F/8F_FdHR1_16bit_stack.tif]

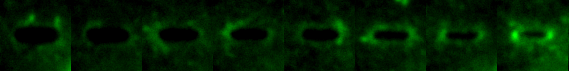

Supplement: Supplementary file 16 — Source data Fig. 8 [file 44318_2025_677_MOESM16_ESM.zip › Figure 8/8F/8F_K166A.tiff]

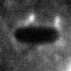

Supplement: Supplementary file 16 — Source data Fig. 8 [file 44318_2025_677_MOESM16_ESM.zip › Figure 8/8F/8F_K166A_16bit_stack.tif]

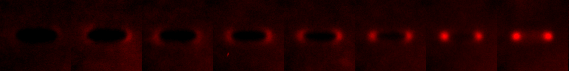

Supplement: Supplementary file 16 — Source data Fig. 8 [file 44318_2025_677_MOESM16_ESM.zip › Figure 8/8G/8G_FBP17.tiff]

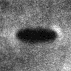

Supplement: Supplementary file 16 — Source data Fig. 8 [file 44318_2025_677_MOESM16_ESM.zip › Figure 8/8G/8G_FBP17_16bit_stack.tif]

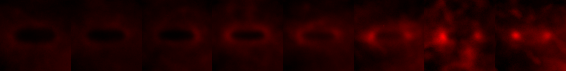

Supplement: Supplementary file 16 — Source data Fig. 8 [file 44318_2025_677_MOESM16_ESM.zip › Figure 8/8G/8G_FdHR1.tiff]

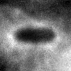

Supplement: Supplementary file 16 — Source data Fig. 8 [file 44318_2025_677_MOESM16_ESM.zip › Figure 8/8G/8G_FdHR1_16bit_stack.tif]

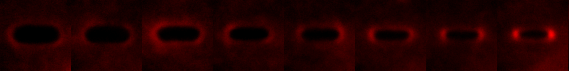

Supplement: Supplementary file 16 — Source data Fig. 8 [file 44318_2025_677_MOESM16_ESM.zip › Figure 8/8G/8G_K166A.tiff]

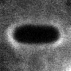

Supplement: Supplementary file 16 — Source data Fig. 8 [file 44318_2025_677_MOESM16_ESM.zip › Figure 8/8G/8G_K166A_16bit_stack.tif]

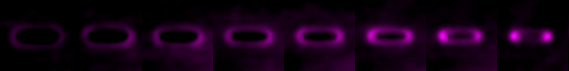

Supplement: Supplementary file 16 — Source data Fig. 8 [file 44318_2025_677_MOESM16_ESM.zip › Figure 8/8H/Figure 8H_FBP17.tiff]

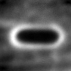

Supplement: Supplementary file 16 — Source data Fig. 8 [file 44318_2025_677_MOESM16_ESM.zip › Figure 8/8H/Figure 8H_FBP17_16bit_stack.tif]

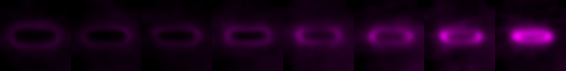

Supplement: Supplementary file 16 — Source data Fig. 8 [file 44318_2025_677_MOESM16_ESM.zip › Figure 8/8H/Figure 8H_FdHR1.tiff]

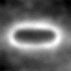

Supplement: Supplementary file 16 — Source data Fig. 8 [file 44318_2025_677_MOESM16_ESM.zip › Figure 8/8H/Figure 8H_FdHR1_16bit_stack.tif]

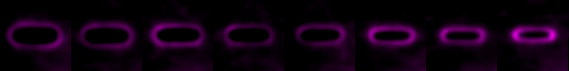

Supplement: Supplementary file 16 — Source data Fig. 8 [file 44318_2025_677_MOESM16_ESM.zip › Figure 8/8H/Figure 8H_K166A.tiff]

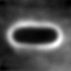

Supplement: Supplementary file 16 — Source data Fig. 8 [file 44318_2025_677_MOESM16_ESM.zip › Figure 8/8H/Figure 8H_K166A_16bit_stack.tif]
