# Supplementary material for: Membrane curvature initiates Cdc42-FBP17-N-WASP clustering and actin nucleation
Source: EMBO J. 2026 Jan 3;45(3):953–86. doi: 10.1038/s44318-025-00677-w (PMC12864879; doi:10.1038/s44318-025-00677-w)
Supplement: Supplementary file 17 — Expanded View Figures [file 44318_2025_677_MOESM17_ESM.pdf]

Expanded View Figures

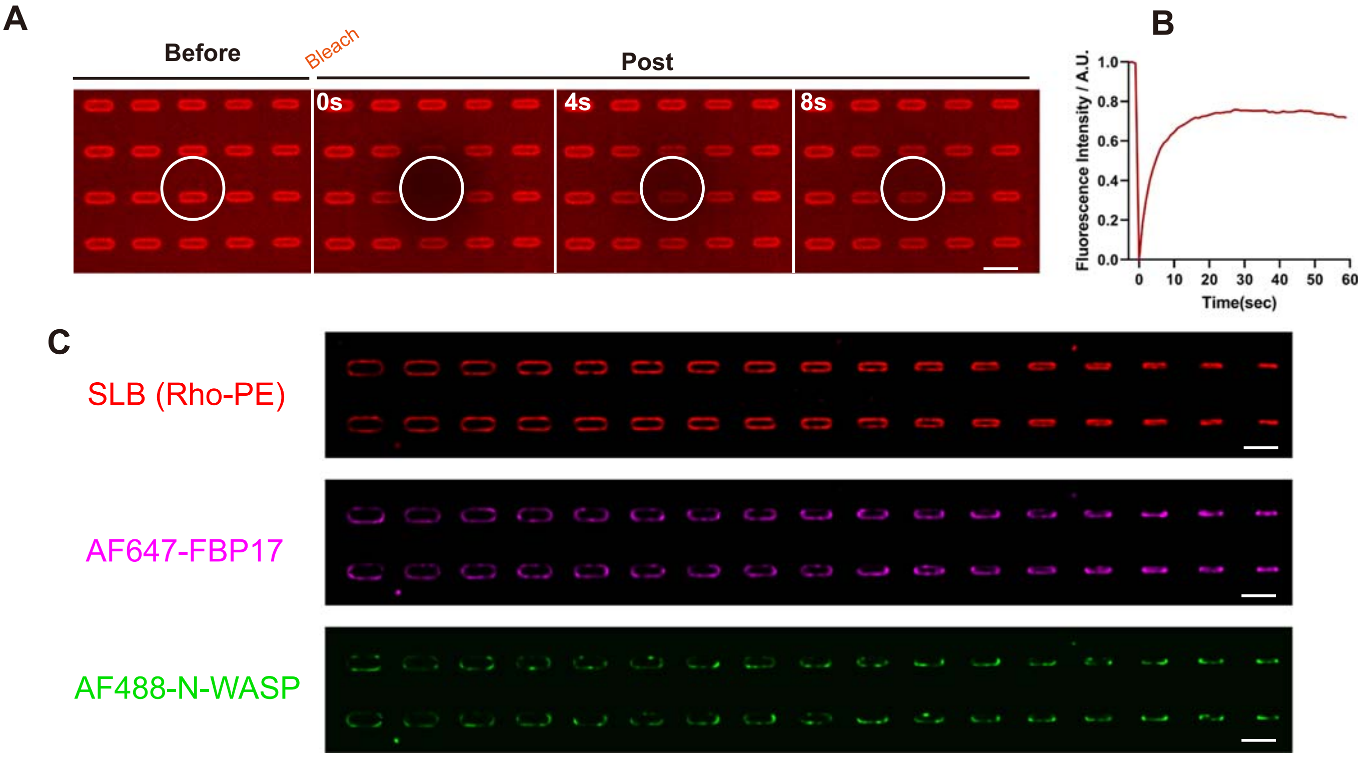

Figure EV1. Curvature-dependent recruitment of FBP17 and N-WASP to membrane nanotubes.

(**A**) Representative FRAP images showing lipid diffusion dynamics on SLBs (10% POPS: 88.5% POPC: 1% PI(4,5)P2:0.5% Rhodamine-PE). White circles indicate the bleached region. Scale bar: 5 µm. (**B**) Quantification of the normalized fluorescence recovery plot of SLB in (**A**). Data were normalized fluorescence intensities (F/F_0). (**C**) Preaveraged confocal microscopy images of SLB, AF647-FBP17 and AF488-N-WASP signal on curved SLB, 200–1000 nm bar width with 50 nm interval. Scale bars: 2 µm.

© The Author(s)

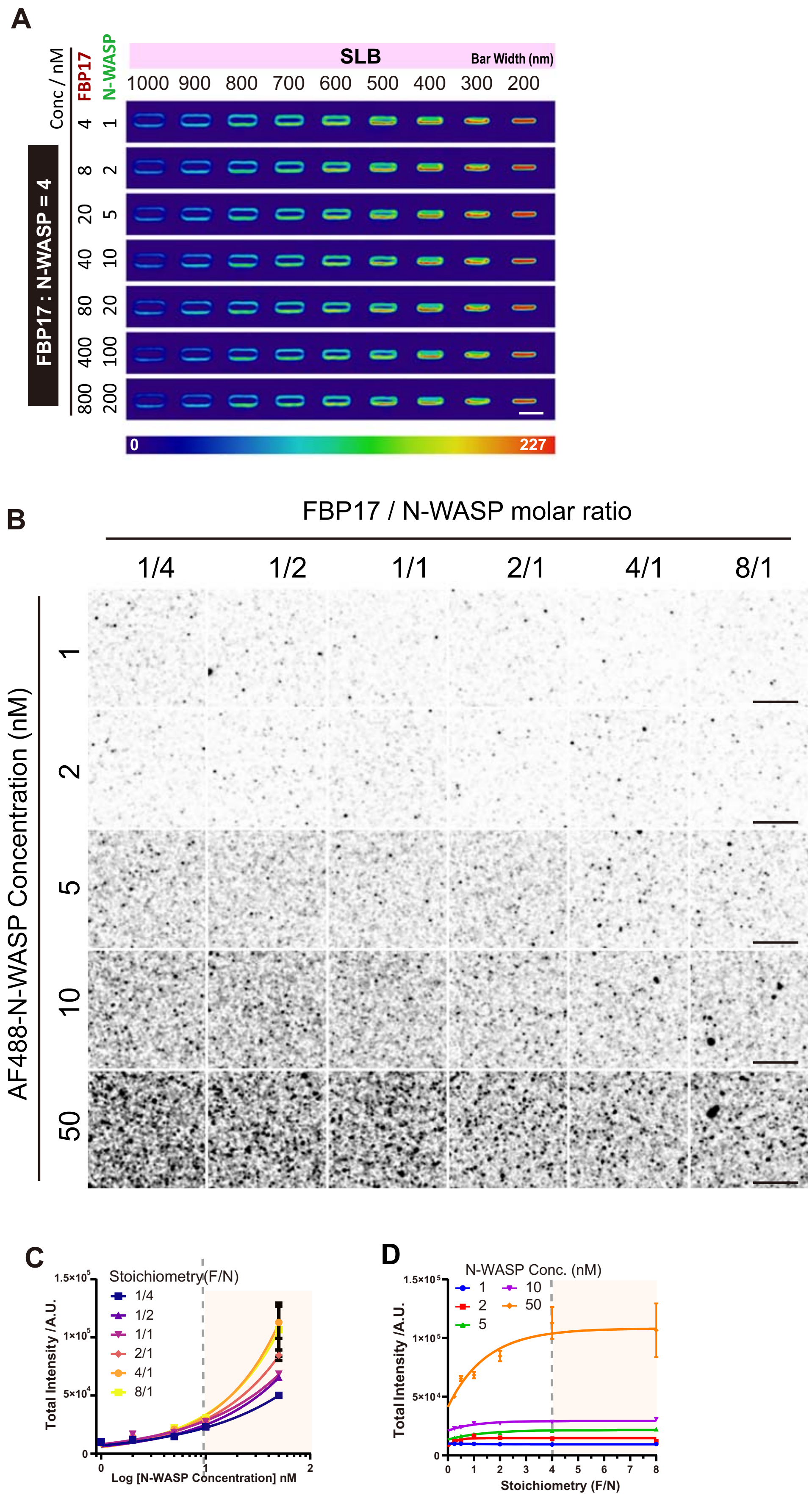
A
Conc / nM
FBP17
N-WASP
FBP17 : N-WASP = 4
SLB
Bar Width (nm)
1000 900 800 700 600 500 400 300 200
4 1
8 2
20 5
40 10
80 20
400 100
800 200
0
227
B
FBP17 / N-WASP molar ratio
1/4 1/2 1/1 2/1 4/1 8/1
AF488-N-WASP Concentration (nM)
1
2
5
10
50
C
Stoichiometry(F/N)
1/4
1/2
1/1
2/1
4/1
8/1
Total Intensity /A.U.
Log [N-WASP Concentration] nM
D
N-WASP Conc. (nM)
1
2
5
10
50
Total Intensity /A.U.
Stoichiometry (F/N)

Figure EV2. N-WASP forms condensate with FBP17 in a concentration- and stoichiometry-dependent manner.

(**A**) Heatmap representation of fluorescence intensities from SLBs at different bar widths (200–1000 nm). Each row corresponds to a fixed FBP17:N-WASP molar ratio of 4:1. Scale bar: 2 µm. (**B**) TIRFM single particle images of AF488-N-WASP at 1, 2, 5, 10, 50 nM on SLB with various stoichiometry of FBP17. Scale bar: 2 µm. (**C**) Plot of single particle intensity of the N-WASP as a function of concentration on flat SLB in (**B**). Lines are binding curves fitted with the Hill equation. $N = 1000$ from three repeated experiments, mean ± SEM are shown. (**D**) Total intensity plot of N-WASP single particles on SLB as a function of F/N stoichiometry on flat SLB in (**B**). $N = 1000$ from three biological repeats, mean and SEM are shown.

© The Author(s)

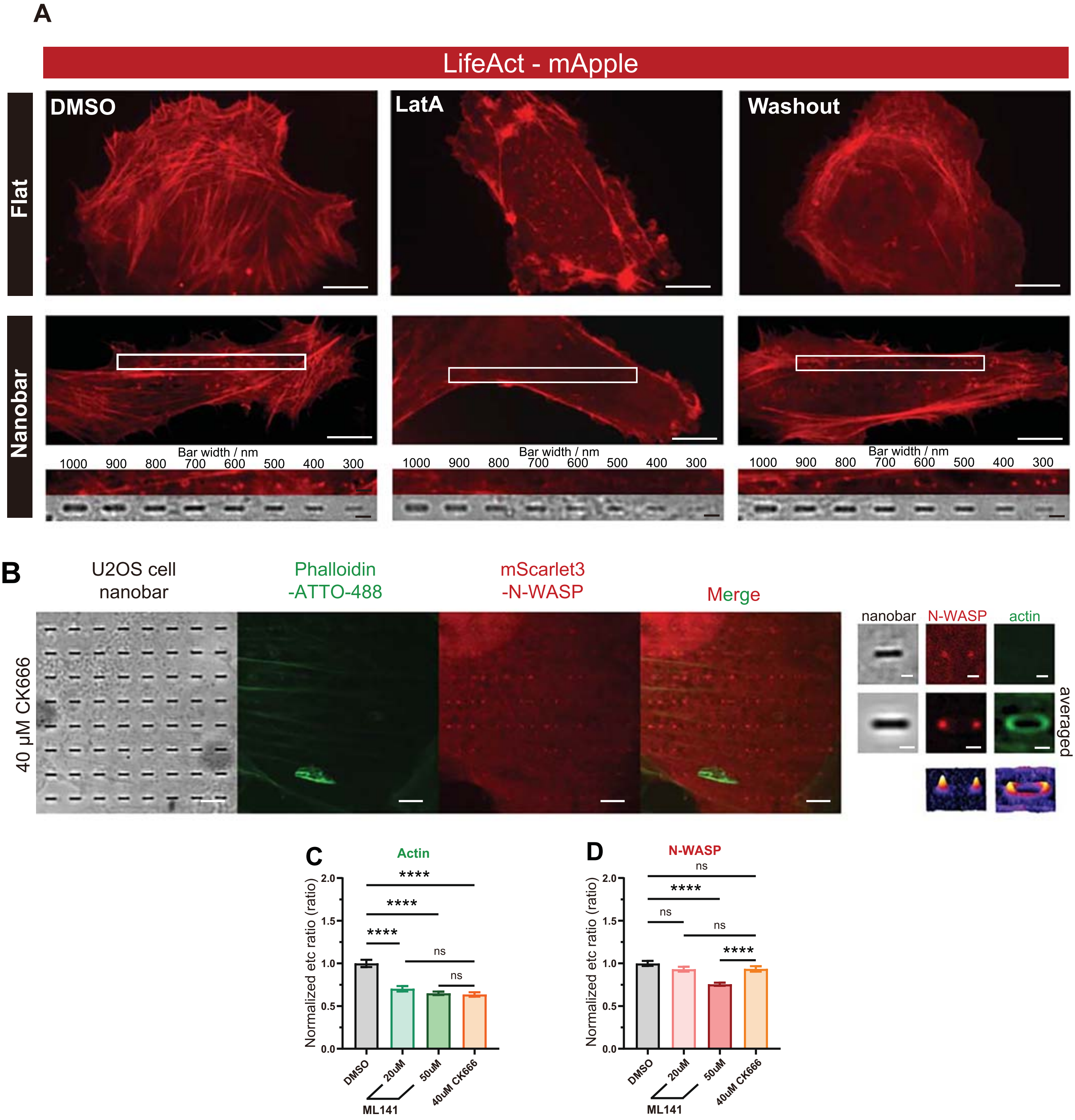

Figure EV3. Actin dynamics and curvature sensing on flat and nanobar substrates.

(**A**) Representative confocal images of Lifeact-mApple-labeled actin filaments in cells treated with DMSO (control), LatA, or after LatA washout, cultured on flat surfaces (top row) or nanobar arrays (bottom row). White boxes in nanobar images indicate regions of interest (ROIs) corresponding to bar widths ranging from 300 to 1000 nm. Scale bars: 10 µm. Zoomed-in views of nanobar ROIs, highlighting actin alignment and curvature adaptation across different bar widths (300–1000 nm). (**B**) Original and averaged confocal images of U2OS fixed cells cultured on 300-nm-wide nanobars with the expression of mScarlet3-N-WASP and the staining with Phalloidin-ATTO-488. Cells were fixed after a 1 h treatment with 40 µM CK666. Scale bar, 10 µm (left) and 1 µm (right). Contrast(averaged): 12–30. (**C**) Normalized Actin signal end-to-center ratio at nanobars 300 nm in width. Sample sizes for treatments under DMSO, 20 or 50 µM ML141, 40 µM CK666 were $N = 634$, 324, 689, and 425 nanobar ends, respectively. Each data point represents the mean ± SEM. (**D**) Normalized mScarlet-N-WASP signal end-to-center ratio at nanobars 300 nm in width. Sample sizes for treatments under DMSO, 20 or 50 µM ML141, 40 µM CK666 were $N = 634$, 324, 689, and 425 nanobar ends, respectively. Each data point represents the mean ± SEM. Statistical analysis was performed using one-way ANOVA followed by Tukey's multiple comparisons test (ns $p > 0.05$, ****$p < 0.0001$).

 © The Author(s)

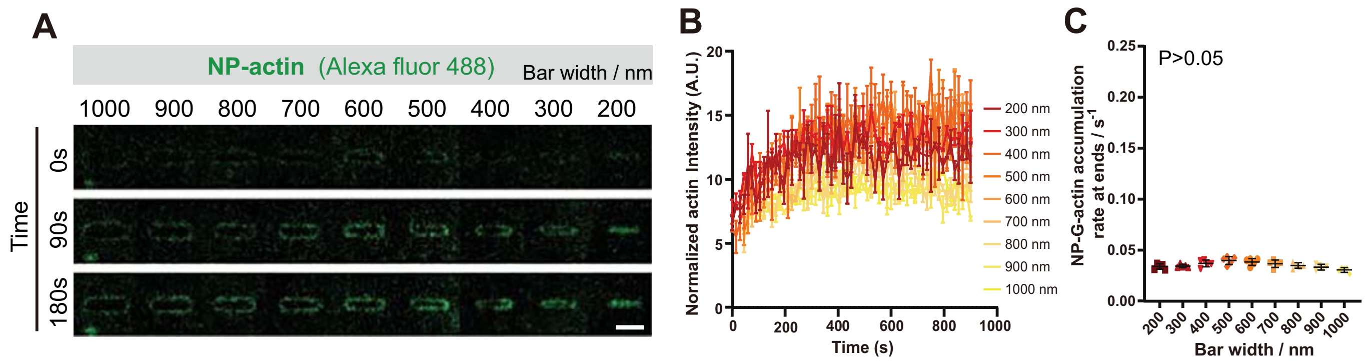

Figure EV4. Curvature-dependent actin assembly in vitro.

(**A**) Averaged confocal images of in vitro reconstitution of 1.5 µM NP-actin (30% AF488 labeled) recruitment at 0/90/180 s on the bilayer at nanobars of 200–1000 nm width with the presence of 200 nM FBP17, 50 nM N-WASP, and 5 nM Arp2/3. Scale bar, 2 µm. (**B**) Normalized signal density of NP-actin based on their corresponding lipid bilayer intensity. Each point represents mean ± SEM from over 15 nanobars. (**C**) NP-G-actin accumulation rate at nanobars of 200–1000 nm width. (The linear fitted slope within 105–300 s range in **B**). Each point represents mean ± SEM from over 15 nanopillars.

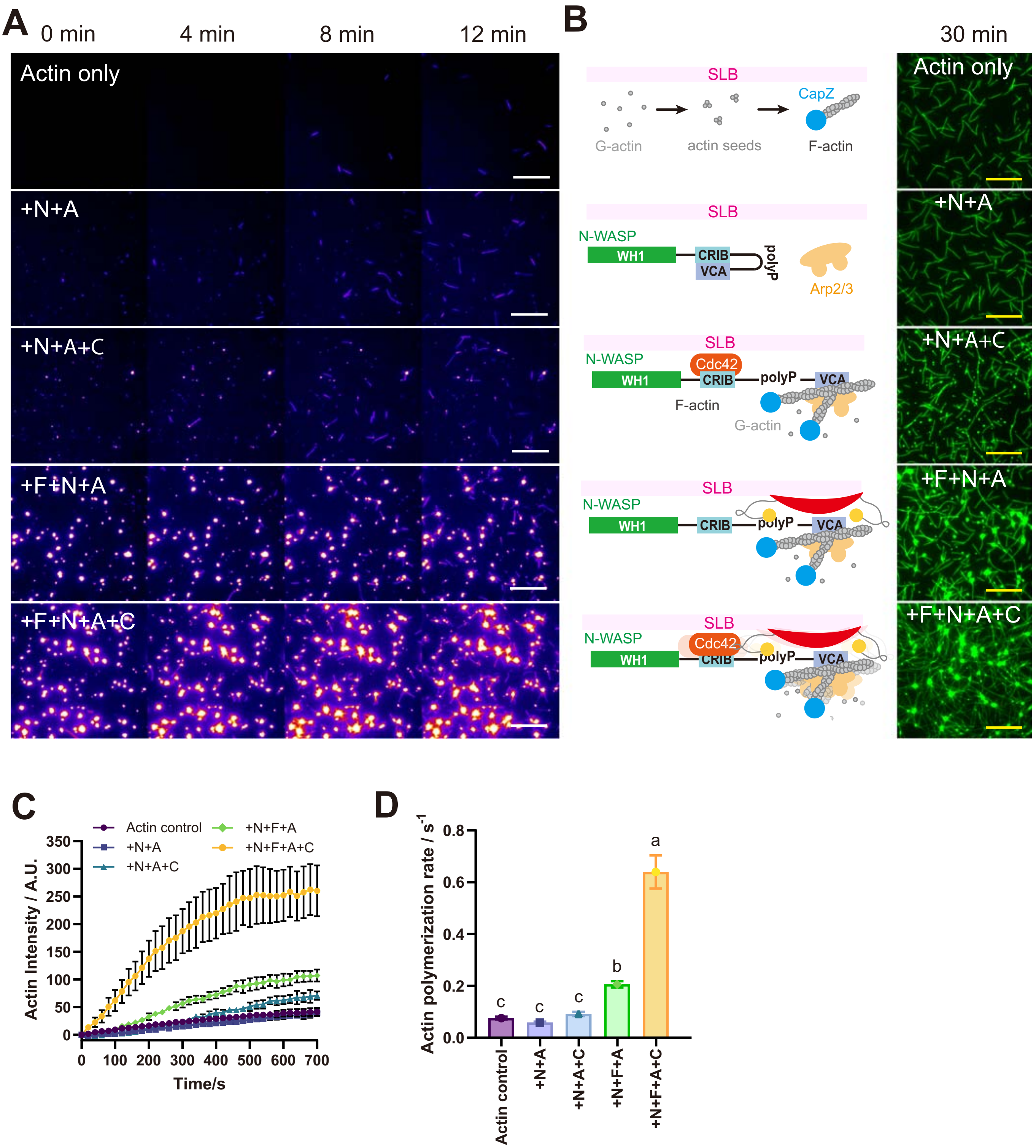

Figure EV5. Synergistic effects of FBP17, N-WASP, and Cdc42 on actin polymerization with actin elongation inhibitor CapZ.

(**A**) Time-lapse fluorescence microscopy images of actin polymerization under different reaction conditions: actin only (control), +N + A, +N + A + C, +F + N + A, and +F + N + A + C. Scale bar: 10 µm. (**B**) Right: Schematic representation of the experimental setup. The schematics illustrate actin polymerization on SLBs in different combinations of actin regulators (N-WASP, FBP17, Cdc42, Arp2/3, and CapZ). Left: Representative endpoint images (30 min) for each condition are shown. Scale bar: 10 µm. (**C**) Quantification of mean actin fluorescence intensity over time in (**A**). (**D**) Actin polymerization rates: the linear fitted slope within 105–350 s range in (**C**). $N = 9$. Each point represents mean ± SEM. Statistical analysis was performed using one-way ANOVA followed by Tukey's multiple comparisons test. Letters (a, b, c) denote statistical significance groups ($p < 0.05$).

 © The Author(s)

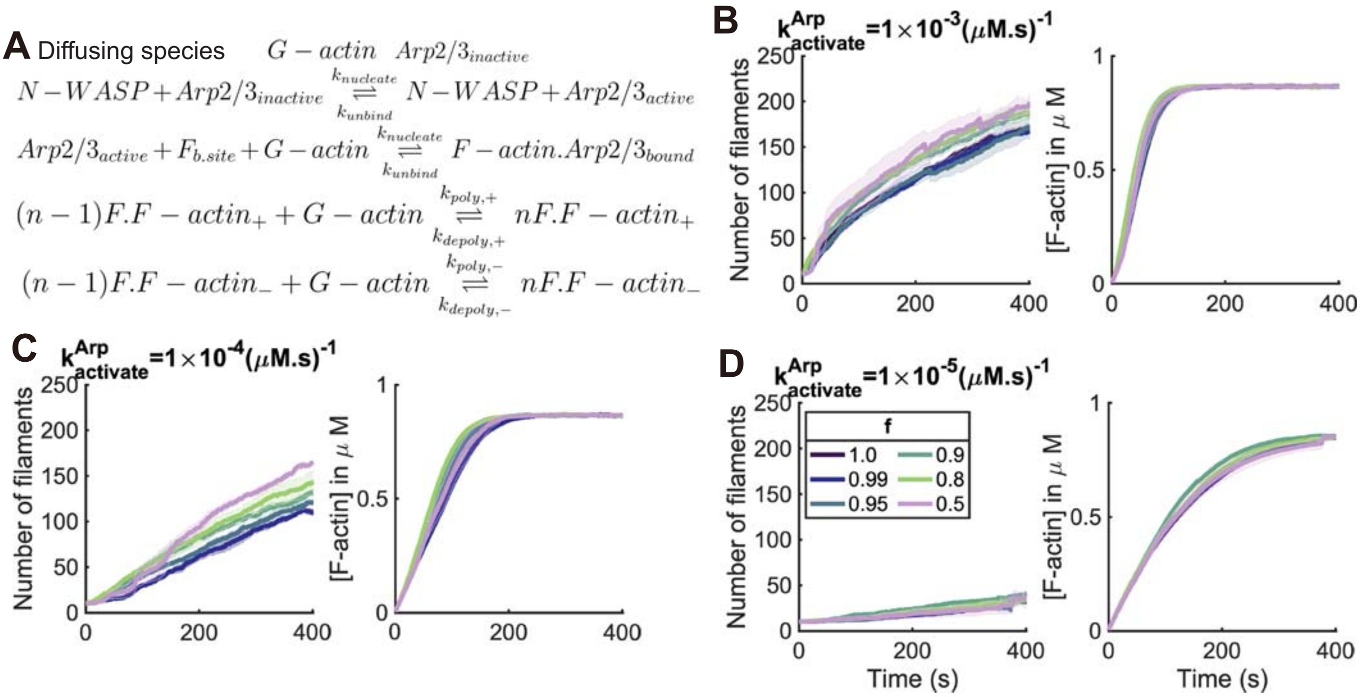

Figure EV6. Spatial localization of N-WASP and N-WASP-driven activation rates are critical to enhance localized nucleation.

(**A**) The set of chemical reactions considered in MEDYAN is shown. G-actin and inactive Arp2/3 are allowed to diffuse throughout the reaction volume. Arp2/3 is activated proportional to the local N-WASP concentration. Active Arp2/3 can bind to a binding site of F-actin (one per ten monomers in this study) to nucleate a new offspring filament. In addition, (de)polymerization reactions at both the plus and minus ends of filaments are considered. (**B–D**) The activation rate of Arp2/3 plays a critical role in controlling the cooperative nucleation process. Plots show mean and standard error of mean plots at various values of localization factor (f) as time series. Each row corresponds to a particular activation rate value.

© The Author(s)

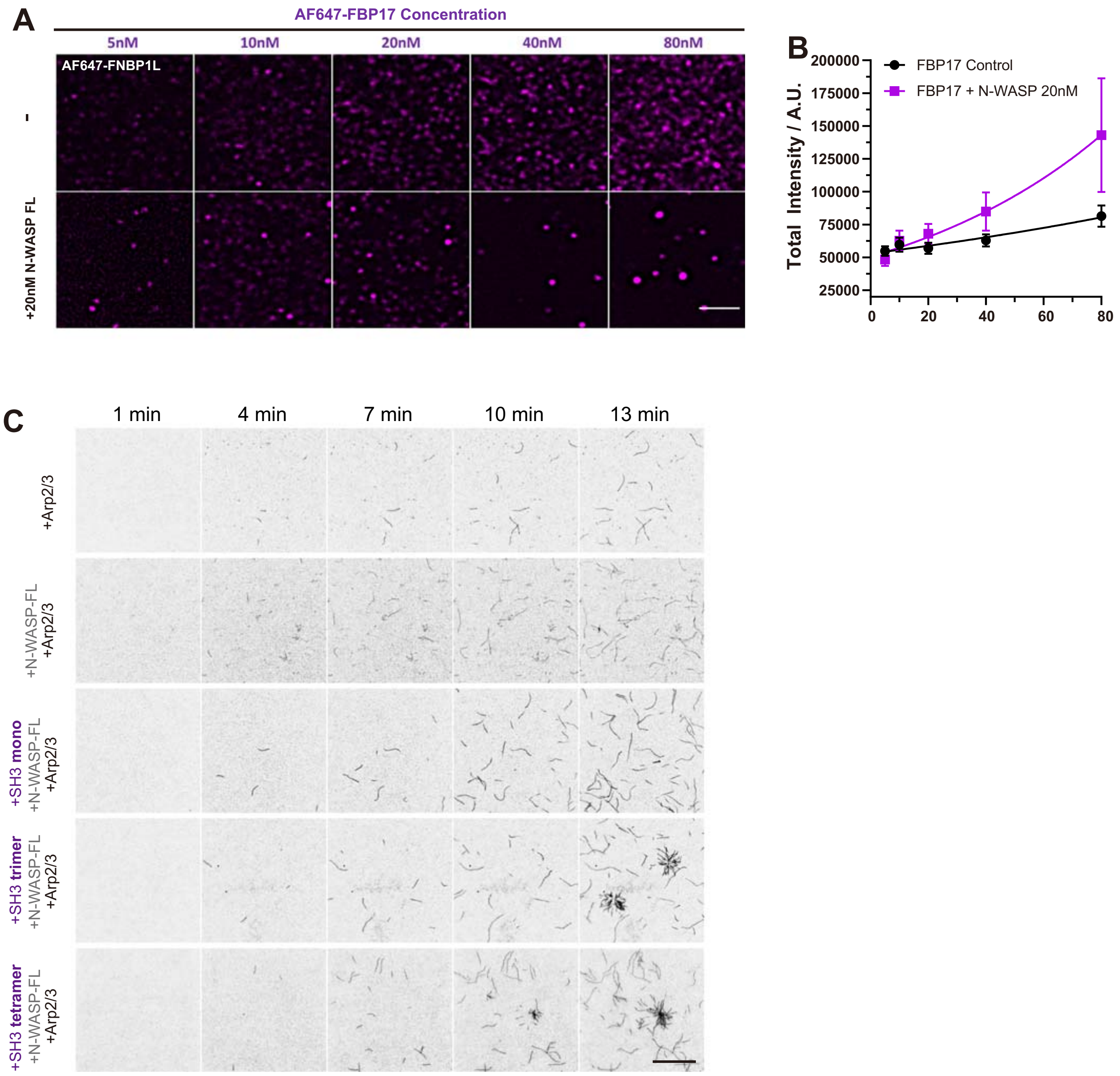

Figure EV7. AF647-FBP17 single particle images and TIRF actin assembly assay of engineered SH3^{FBP17}.

(**A**) TIRFM single particle images of AF647-FBP17 at 5, 10, 20, 40, 80 nM on SLB in the absence(upper)/presence(bottom) of 20 nM N-WASP FL. Scale bar, 5 μm. (**B**) Quantification of total fluorescence intensity of FBP17 as a function of concentration, with or without N-WASP in (**B**). (**C**) TIRF images of actin polymerization (10% Oregon-labeled) with different combinations of 80 nM FBP17/oTri-SH3/oTet-SH3, 20 nM N-WASP FL and 5 nM Arp2/3. Scale bar, 10 μm.

 © The Author(s)
